# Antigen-Presenting B Cells Program the Efferent Lymph T Helper Cell Response

**DOI:** 10.3389/fimmu.2022.813203

**Published:** 2022-03-09

**Authors:** Samuel Alsén, Jakob Cervin, Yaxiong Deng, Louis Szeponik, Ulf Alexander Wenzel, Joakim Karlsson, Helena Cucak, Megan Livingston, David Bryder, Qianjin Lu, Bengt Johansson-Lindbom, Ulf Yrlid

**Affiliations:** ^1^ Department of Microbiology and Immunology, Institute of Biomedicine, University of Gothenburg, Gothenburg, Sweden; ^2^ Sahlgrenska Center for Cancer Research, Department of Surgery, University of Gothenburg, Gothenburg, Sweden; ^3^ Immunology Section, Lund University, Lund, Sweden; ^4^ Department of Dermatology, Second Xiangya Hospital, Central South University, Hunan Key Laboratory of Medical Epigenomics, Changsha, China; ^5^ Harry Perkins Institute of Medical Research, University of Western Australia, Perth, WA, Australia; ^6^ Division of Molecular Hematology, Lund University, Lund, Sweden; ^7^ Immunological Memory Group, Department of Health Technology, Technical University of Denmark, Kgs. Lyngby, Denmark

**Keywords:** efferent lymph, T cells, B cells, gut-homing CD4^+^ T cells, small intestinal lamina propria

## Abstract

B cells interact with T follicular helper (Tfh) cells in germinal centers (GCs) to generate high-affinity antibodies. Much less is known about how cognate T–B-cell interactions influence Th cells that enter circulation and peripheral tissues. Therefore, we generated mice lacking MHC-II expressing B cells and, by thoracic duct cannulation, analyzed Th cells in the efferent lymph at defined intervals post-immunization. Focusing on gut-draining mesenteric lymph nodes (MLNs), we show that antigen-specific α_4_β_7_^+^ gut-homing effector Th cells enter the circulation prior to CXCR5^+^PD-1^+^ Tfh-like cells. B cells appear to have no or limited impact on the early generation and egress of gut-homing Th cells but are critical for the subsequent appearance of Tfh-like cells that peak in the lymph before GCs have developed. At this stage, antigen-presenting B cells also reduce the proportion of α_4_β_7_^+^ Th cells in the MLN and efferent lymph. Furthermore, cognate B-cell interaction drives a broad transcriptional program in Th cells, including IL-4 that is confined to the Tfh cell lineage. The IL-4-producing Tfh-like cells originate from Bcl6^+^ precursors in the LNs and have gut-homing capacity. Hence, B cells program the efferent lymph Th cell response within a limited window of time after antigenic challenge.

## Introduction

Activation of naive CD4^+^ T helper cells (Th) leads to proliferation and differentiation into peripheral T effector (Teff) cells and lymph node (LN)-resident T follicular helper (Tfh) cells (1, 2). While the Teff cells exit the LN and enter the circulation through the efferent lymphatic system, Tfh cells underpin B-cell expansion and affinity maturation within germinal centers (GC). The two branches of Th cells develop in parallel under the control of the transcriptional repressors Bcl6 and Blimp-1, which act as reciprocal antagonists to ensure the development of largely mutually exclusive subsets (3–7). This layer of transcriptional regulation acts in concert with other lineage-specific transcription factors to further specify the Teff cell fate commitment. Dendritic cells (DCs) have been shown to have non-redundant roles in the differentiation toward, for example, the Th1 and Th17 effector cell subsets (8, 9). Antigen presentation by B cells instead represents an essential step for the appearance of fully committed Tfh cells (3, 10, 11). In the absence of B cells, or in mice lacking MHC class II (MHC-II) selectively on B cells, T cells thus fail to acquire a high-level expression of PD-1 and Bcl-6, phenotypic traits of *bona fide* Tfh cells residing within GCs (GC-Tfh) [reviewed in (12)]. In contrast to their well-documented role as antigen-presenting cells (APCs) during the final step of Tfh cell commitment, less is known regarding how B cells influence the peripheral Th cell compartment. Yet, several studies have indicated that B cells can have important functions distinct from humoral immunity in both protective and autoimmune responses (13, 14). This suggests that antigen presentation by B cells can have bearing also on Th cells seeking peripheral tissues. It, however, remains to be determined if and to what extent B cells alter the composition and dynamics of the efferent lymph Th cell response.

Circulating CXCR5 and PD-1 expressing “Tfh-like cells” have been described in several studies [reviewed in (15)] and proposed to represent immediate precursors of GC-Tfh cells (16). Consistent with this idea, an increased proportion of activated Tfh-like cells in the blood of humans 7–10 days immunization correlates with antibody responses to influenza vaccination (17). Steady-state human efferent lymph has also been shown to contain Tfh-like cells with some GC-Tfh cell characteristics (18), and recently, Tfh cells in human tonsils and Tfh-like cells in blood were shown to be clonally related but distinct from non-Tfh cells (19). While these studies collectively indicate that Tfh-like cells in the blood share ontogeny with GC-Tfh cells in the LN, it remains elusive if the peripheral subset develops as a consequence of cognate interactions with B cells (16). It is also unclear if these Tfh-like cells, similar to the peripheral Teff cell subsets, have the ability to enter peripheral tissues.

Previous studies in skin- and gut-draining LNs have shown that the lineage decision between Teff and Tfh cells, distinguished by the exclusionary expression of CXCR5 and peripheral homing receptors, is an early event (1, 2). The majority of Teff cells generated in the mesenteric LNs (MLNs) display high levels of the gut-homing-associated integrin α_4_β_7_ (20). To address antigen presentation by B cells in relation to development and LN exit of Teff and Tfh-like cells, we have collected thoracic duct leukocytes (TDLs) from immunized wild-type and mixed bone marrow chimeric mice in which B cells lack MHC-II molecules (MHCII^B−/−^). We show that antigen-specific α_4_β_7_^+^ Teff cells enter the efferent lymph with a faster kinetics relative to CXCR5^+^PD-1^+^ Tfh-like cells. The Tfh-like cells peak in the lymph around day 5 post-immunization (p.i.) but are absent at the time when GCs are fully established. Antigen presentation by B cells is required for the generation of Tfh-like cells and also counteracts a sustained peripheral α_4_β_7_^+^ Teff cell response. In addition, T–B-cell interaction drives a broad transcriptional program in the Th cell where IL-4 is confined to the Tfh cell lineage. By using a transgenic reporter for IL-4 secretion, we have tracked the Tfh-like cells in the efferent lymph and as they enter the intestinal mucosa. This reveals a previously unidentified role of B cells in regulating the generation of peripheral Teff and circulatory Tfh-like cells.

## Material and Methods

### Mice

All mice were housed in specific pathogen-free conditions at the Experimental Biomedicine animal facility in Gothenburg or at the Biomedical Center animal facility, Lund, Sweden. C57BL/6 (WT) mice were bought from Taconic (Denmark). CD45.1^+^ OT-II mice were generated by crossing OT-II (21) × C57BL/6-CD45.1^+^. μMT mice (22), MHCII^−/−^ mice (23), and CD45.1^+^ OT-II mice, all on C57BL/6 background, were all bred in-house. KN2xOT-II mice were generated by crossing OT-II mice onto KN2/KN2 (24) mice (kindly provided by Marcus Svensson Frej, Lund University, Sweden). CD19-Cre (25) mice were crossed with MHCII^fl/+^ (23) mice to generate CD19-Cre × MHCII^fl/+^ mice, which in turn were bred with MHCII^fl/+^ mice to generate experimental mice.

### Bone Marrow Chimeras

Bone marrow cells from donor mice were flushed from the tibia and femur, red blood cells were lysed using BD Pharm Lyse (BD Biosciences, USA), and the remaining cells were filtered and resuspended in PBS. Eight- to 12-week-old recipient mice were irradiated with 8 Gy using RS-2000 irradiator (RadSource, USA) prior to receiving 10^6^ bone marrow cells by i.v. injection to generate control (μMT/C57BL/6) or MHCII^B−/−^ (μMT/MHCII^−/−^) chimeras as previously described (26). Chimerism was confirmed by flow cytometry analysis of splenocytes at the time of sacrifice.

### Adoptive Transfer and Immunizations

CD4^+^ T cells were enriched from spleens from OT-II mice by positive selection using CD4 MicroBeads (Miltenyi Biotec) and MACS LS column (Miltenyi Biotec, Germany). Viability was assessed by Trypan blue (Gibco, USA) staining and 2 × 10^5^ cells were transferred into recipients by i.v. injection. Mice were immunized the following day by i.p. injection of 300 μg ovalbumin (OVA, Sigma-Aldrich, USA) and 100 μg high molecular weight pI:C (Invivogen, USA) or 2 μg cholera toxin (CT, Sigma-Aldrich, USA) in PBS. Mice immunized orally were gavaged with 300 μg OVA and 10 μg CT in 3% NaCHO_3_.

### Thoracic Duct Cannulation

Thoracic duct cannulation procedures of mice were performed as described in rats (27), with minor modifications to accommodate for differences in size between species. In short, 8–12-week-old mice that had been adoptively transferred with OT-II or KN2xOT-II cells i.v. and subsequently immunized with OVA + polyI:C i.p. were gavaged with 0.2 ml rape seed oil to visualize the lymphatics. The mice were anesthetized with isoflurane and the thoracic lymph duct was cannulated by the insertion of a polyurethane cannula (2Fr; Linton Instrumentation). The lymph was collected in PBS supplemented with 24 U/ml heparin and 12 mM EDTA on ice for 16–24 h. During surgery, the mice received analgesic treatment by subcutaneous administration of Rimadyl (Orion Pharma Animal Health, Finland) and Temgesic (Indivior, USA) as well as intradermal administration of Marcain (Aspen Nordic, Ireland) in the wound area. After overnight collection of TDL, animals were sacrificed and MLNs harvested.

### Handling of Cells

Organs were mechanically disrupted into single-cell suspensions in PBS supplemented with 2% fetal bovine serum (FBS, Gibco) and 2 mM EDTA (Sigma-Aldrich) and passed through cell strainers. TDLs were washed once in FACS buffer before being stained for flow cytometric analysis. Tubes for collection of blood samples were weighed prior to and after collection of terminal blood. Whole blood was subjected to red blood cell lysis and washed before analysis of the cells.

### Isolation of Lymphocytes From Lamina Propria

Mesenteric fat and Peyer’s patches were removed from the small intestine. After rinsing the small intestine with PBS to remove feces and excessive mucus, the remaining tissue was cut into small pieces. After washing the tissue in Ca^2+^/Mg^+^-free Hanks’ Balanced Salt Solution (HBSS, Gibco) supplemented with 2% FBS and 1 mM 4-(2-hydroxyethyl)-1-piperazineethanesulfonic acid (HEPES, Sigma-Aldrich), the tissue was incubated at 37°C for 15 min while being magnetically stirred in Ca^2+^/Mg^+^-free HBSS media containing 1 mM HEPES, 2% horse serum (Fisher Scientific, USA), and 2 mM EDTA for three rounds. After washing in normal HBSS, the tissue was digested by 50 μg/ml Liberase TM (Roche, Switzerland) in RPMI 1640 basal media (Gibco) supplemented with 10% fetal bovine serum, 1 mM HEPES, and 1 mM sodium pyruvate (Sigma-Aldrich) for 30 min at 37°C. Digested tissue was mashed over a nylon mesh and washed with PBS, and lymphocytes were isolated by density separation using 40%/70% layers of Percoll (GE Healthcare, USA).

### Flow Cytometry

To exclude dead cells, either propidium iodide (Biolegend, USA), 7-amino-actinomycin D (eBioscience, USA), or Live/Dead Aqua (Invivogen) was used. The following antibodies were used for surface staining: α_4_β_7_ (DATK32), B220 (RA3-6B2), CD4 (GK1.5), CD45.2 (104), and PD-1 (J43) from eBioscience; CCR9 (CW-1.2), CD4 (RM4-5), CD44 (IM7), CD45.1 (A20), CD62L (MEL-14), human CD2 (TS1/8), I-A/I-E (M5/114.15.2), and PD-1 (29F.1A12) from BioLegend; Bcl6 (K112-91), CXCR5 (2G8), IL-4 (11B11), IL4Rα (mIL4R-M1), mouse IgG1 k isotype, rat IgG1 isotype, and streptavidin-conjugated fluorochromes from BD Biosciences, USA; and donkey F(ab)_2_ anti-rat H+L Alexa647 from Jackson ImmunoResearch Laboratories, USA. Cell surface staining was performed for 20 min at 4°C or 45 min at 4°C for CXCR5 and donkey anti-rat antibodies. Prior to intracellular staining for IL-4, MLN cells were incubated at 37°C and 5% CO_2_ in complete RPMI 1640 media for 4 h in the presence of 50 ng/ml PMA, 500 ng/ml ionomycin and, for the last 3 h, 10 µg/ml brefeldin A (all from Sigma-Aldrich). Intracellular staining of Bcl6 and IL-4 was performed using the FoxP3/Transcription factor staining buffer set (eBioscience) according to the manufacturer’s instructions. Flow cytometry data were acquired on FACSAria III (BD Biosciences) or LSRII (BD Biosciences) instruments and analyzed using FlowJo software (Tree Star).

### Immunofluorescence

Tissue was frozen in OCT (Histolab, Sweden) by placing it first in isopentane and subsequently in liquid nitrogen. Cryosections of 7 µm thickness (Leica Microsystems, Germany) were fixed with 50% acetone at RT for 30 s, directly followed by 100% acetone on ice for 10 min. Sections were air-dried for 10 min. Blocking of endogenous peroxidase was performed with PeroxAbolish (Histolab) for 1 h followed by avidin/biotin blocking for 10 min each (SIG-31126, Biolegend). Incubation with primary antibodies against CD45.2-FITC (1:200, 104, Biolegend), hCD2-biotin (1:100, RPA-2.10, Biolegend), CD3-AF594 (1:300, 17A2, Biolegend), IgD-PerCP (1:100, 11-26c.2a, Biolegend), and KI67-rabbit (1:100, ab16667, Abcam, UK) was performed for 1 h at RT. Next, sections were incubated with anti-rabbit-BV480 (1:100, 564879, BD) for 40 min at RT, and streptavidin-HRP (B40932, Thermo Fisher) was added for the last 15 min. Signal for hCD2 was developed with the Cy3 Tyramide kit (SAT704A001EA, Perkin Elmer, USA) using 1:1,000 tyramide for 5 min. Sections were stained with DAPI for 10 min (Thermo Fisher) and mounted with ProLong Diamond Antifade (Thermo Fisher). Tissue sections were imaged on Metafer Slide Scanning Platform (MetaSystems, Germany) with a SpectraSplit filter set (Kromnigon, Sweden). Pictures were exported from VSViewer (MetaSystems) and black/white levels of the images were adjusted with Fiji ImageJ and Photoshop CC2017 (Adobe, USA).

### Gene Expression Analysis

Total live B220^−^CD4^+^CD45.1^+^ OT-II cells or subsets of OT-II cells based on CD62L and CXCR5 expression levels from spleens and MLN were sorted into RLT Lysis buffer (Qiagen, Germany) supplemented with 1% β-mercaptoethanol (Sigma-Aldrich) and stored at −80°C until further use. mRNA was isolated using RNeasy Micro Kit (Qiagen) according to the manufacturer’s instructions. Conversion of mRNA into cDNA was performed using SuperScript Double-Stranded cDNA Synthesis Kit (Invitrogen, USA) following the manufacturer’s instructions. For the quantification of mRNA transcript levels, cDNA from total OT-II cells was analyzed on a Biomark HD (Fluidigm, USA) or a thermal cycler (Bio-Rad, USA) in triplicates. Primers used were ordered from Fluidigm or Bio-Rad. Converted cDNA from total OT-II cell sorted from the MLN was sent to KFB Regensburg, Germany, for microarray analysis.

### Microarray Preprocessing and Differential Expression Testing

Microarray raw data in CEL format were normalized in R (v. 3.5.3) using the RMA (Robust Multichip Average) algorithm implemented in the “oligo” package (v. 1.46) (28). Probe sets were mapped to gene symbols using the package “mogene11sttranscriptcluster.db” (v. 8.7.0). For each gene, the probe set with the highest average expression across all samples was retained. Genes with a coefficient of variation greater than median were retained for expression tests. Differential expression tests were carried out using the “eBayes” and “ImFit” functions of the “limma” (29) R package (v. 3.38.3). *p*-values were adjusted for multiple testing using the Benjamini–Hochberg method. Differentially expressed genes were visualized with the package “pheatmap” (v. 1.0.12). Gene set enrichment analysis was performed with the STRING (30) database online tool (v. 11.0), using all tested genes and their respective log_2_ fold changes. A protein interaction network corresponding to the genes in the most strongly enriched Gene Ontology (31) “biological process” category was visualized using STRING, based on the evidence classes “Textmining,” “Experiments,” and “Databases,” with the default confidence threshold of 0.4.

### Statistics

Statistical analysis of data was carried out in Prism (GraphPad Software) using one-way ANOVA with Sidak’s post-test for multiple comparison or unpaired two-tailed Student’s *t*-test for groups of two unless otherwise stated. Exact *p*-values below a threshold of *p <*0.05 are reported in graphs.

## Results

### Following Immunization, α_4_β_7_^+^ T Effector Cells Enter Circulation Prior to CXCR5^+^PD-1^+^ Tfh-Like Cells

**Figure 1:**
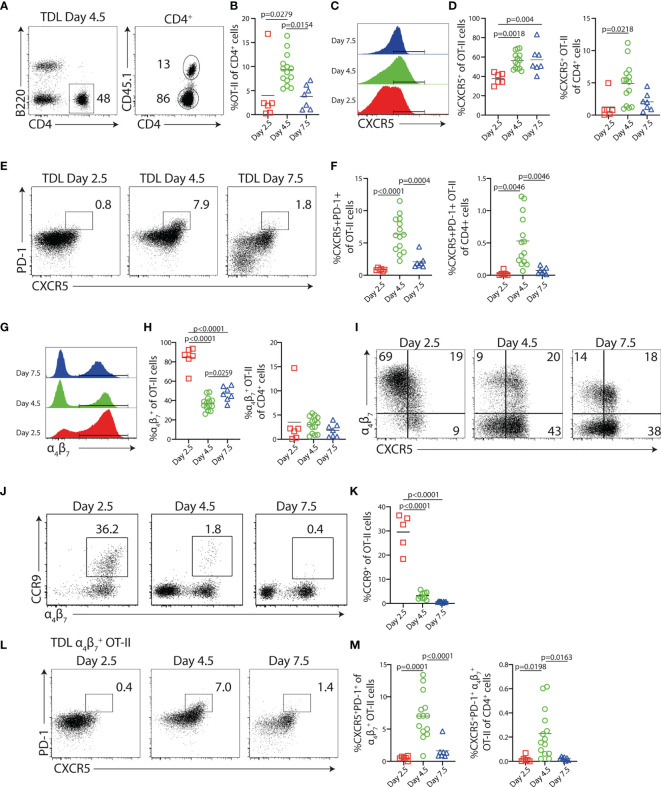
Characterization of Ag-specific Th cells in the efferent lymph following immunization. CD45.1^+^ OT-II cells were transferred into congenic CD45.2^+^ hosts 1 day prior to i.p. immunization with OVA + polyI:C, and thoracic duct leukocytes (TDLs) were collected and analyzed by flow cytometry at indicated intervals. **(A)** Identification of B220^−^ CD4^+^ CD45.1^+^ OT-II cells among TDLs collected 4.5 days p.i. and **(B)** percentage of OT-II among CD4^+^ TDLs at indicated intervals. **(C, D)** Expression of CXCR5 by OT-II TDLs **(C)** and frequency of CXCR5^+^ cells of OT-II TDLs and of CD4^+^ TDLs collected at indicated intervals **(D)**. **(E)** CXCR5 and PD-1 expression by OT-II TDLs and **(F)** frequency of CXCR5^+^PD-1^+^ cells of OT-II cells and of CD4^+^ TDLs. **(G)** Expression of α_4_β_7_^+^ by OT-II TDLs and **(H)** frequency of α_4_β_7_^+^ cells of OT-II TDLs and α_4_β_7_^+^ OT-II cells of CD4^+^ TDLs. **(I)** CXCR5 and α_4_β_7_ expression by OT-II TDLs. **(J)** CCR9 and α_4_β_7_ expression by OT-II TDLs and **(K)** frequency of CCR9^+^ cells of OT-II TDLs. **(L)** Expression of PD-1 and CXCR5 among α_4_β_7_^+^ OT-II TDLs and **(M)** frequency of CXCR5^+^PD-1^+^ cells of α_4_β_7_^+^ OT-II TDLs and of all CD4^+^ TDLs. Data were collected during two to four individual experiments per interval, *n* = 6–14. All graphs show individual data points with mean. One-way ANOVA with Tukey’s multiple comparison test; *p*-values < 0.05 are reported.

In this study, we wanted to define how B cells impact on the pool of Th cells exiting the LN after immunization. We also wished to determine if antigen-presenting B cells are required for the appearance of the previously characterized blood-borne Tfh-like cells and, furthermore, explore if these cells contribute to the peripheral Th cell subsets ultimately entering peripheral tissues, as for example the intestinal mucosa. Therefore, congenic CD4^+^ OT-II cells specific for OVA were adoptively transferred to wt recipients. During 24-h time periods centered around 2.5, 4.5, and 7.5 days p.i. with OVA and polyI:C, efferent lymph TDLs were collected by cannulation (i.e., lymph was collected 48–72, 96–120, or 168–192 h p.i.) (**Supplementary Figure 1A**). CD45.1^+^ OT-II cells were identified in the efferent lymph by flow cytometry (**Figure 1A** **and** **Supplementary Figure 1B**). To account for variability in volume of lymph collected from individual mice, resulting in large variations in total number of cells collected (ranging from 2 to 20 × 10^6^ cells), the frequency of OT-II cells among total CD4^+^ TDLs, rather than absolute cell numbers, was determined by flow cytometry (**Figure 1B**). OT-II cells represented 2.5% of the total CD4^+^ TDLs at day 3, increased to 10% day 5 and then contracted to 5% at day 8 (**Figure 1B**). In comparison to endogenous CD4^+^ TDLs, OT-II cells collected during day 3 uniformly expressed higher levels of CD44 and PD-1 as well as reduced levels of CD62L (**Supplementary Figures 1C–E** **)**. The frequency of OT-II cells expressing CXCR5 increased from 40% to 60% over the two first collection periods to plateau thereafter (**Figures 1C, D** **)**. Given the low output of total OT-II cells during days 3 and 8 after immunization (**Figure 1B**), the time window for when a significant number of CXCR5^+^ antigen-specific T cells entered the lymph was narrow and centered to day 5 p.i. This could be directly illustrated when displaying the frequency of CXCR5^+^ OT-II cells as percentage of all TDL CD4^+^ T cells (**Figure 1D**) and was even more evident for CXCR5^+^PD-1^+^ Tfh-like OT-II cells (**Figures 1E, F** **)**. While a substantial proportion of the lymph-borne OT-II cells expressed the Tfh-like phenotype on day 5 (6.4% ± 2.7%; mean value ± SD), they were dramatically reduced during the earlier and later collection periods (0.9% ± 0.2% and 2.1% ± 1.1% for days 3 and 8, respectively) (**Figures 1E, F** **)**.

In marked contrast to the delayed appearance of Tfh-like cells, 80%–90% of the OT-II cells that exited the LNs during day 3 expressed the gut-homing marker α_4_β_7_. This declined to 40%–45% in the later collections (**Figures 1G, H** **)**. The observation that most TDL OT-II cells express α_4_β_7_ 3 days after i.p. immunization indicates that T-cell expansion in the MLNs to a large extent accounts for the antigen-specific T-cell response detected in the TDL preparations, as this integrin is selectively induced on T cells activated in the intestinal inductive sites (20, 32). α_4_β_7_^+^ OT-II cells collected on day 3 did not present with substantial CXCR5 expression, indicating that the cells represent gut-homing Teff cells developmentally distinct from the Tfh cell lineage (**Figure 1I**). Consistently, a large fraction of these α_4_β_7_^+^ OT-II cells co-expressed the chemokine receptor CCR9 that mediates homing to the small intestinal lamina propria (SI-LP) (**Figures 1J, K** **)** (33). Five days after immunization, α_4_β_7_ was expressed by a significantly lower proportion of exiting OT-II cells and the amount detected per cell basis was also reduced at this time point relative to the gut-homing subset entering the lymph 3 days after immunization. Furthermore, α_4_β_7_ expression was now mostly associated with the cohort of CXCR5-expressing cells, dominating the TDL OT-II cell response at this time, and CCR9 could barely be detected on these cells. Collectively, these findings indicated an overall different nature of the T cells entering the lymph 5 days after immunization and we therefore asked if the α_4_β_7_^+^ CXCR5^+^ OT-II cells appearing in the lymph with a delayed kinetics include Tfh-like cells. Analysis of CXCR5 versus PD-1 expression after gating on α_4_β_7_^+^ OT-II cells indeed revealed the presence of α_4_β_7_^+^ OT cells with the CXCR5^+^PD-1^+^ Tfh-like phenotype. Like the total Tfh-like cell population (**Figures 1E, F** **)**, this subset peaked in the efferent lymph 5 days after immunization and was not present in the earlier or later collections (**Figures 1L, M** **)**.

Collectively, these results establish that CXCR5^+^PD-1^+^ Tfh-like cells enter the lymph with delayed kinetics compared with the CXCR5^−^ α_4_β_7_^+^ gut-homing subset after immunization. Furthermore, exit of the Tfh-like cells from the MLNs, as well as from other secondary lymphoid organs, occurs during a limited period and ceases between days 5 and 8 post-immunization.

### Increased OT-II Cell Precursor Frequency Leads to Enhanced α_4_β_7_ Expression But Does Not Alter the Frequency of CXCR5-Expressing Cells

**Figure 2:**
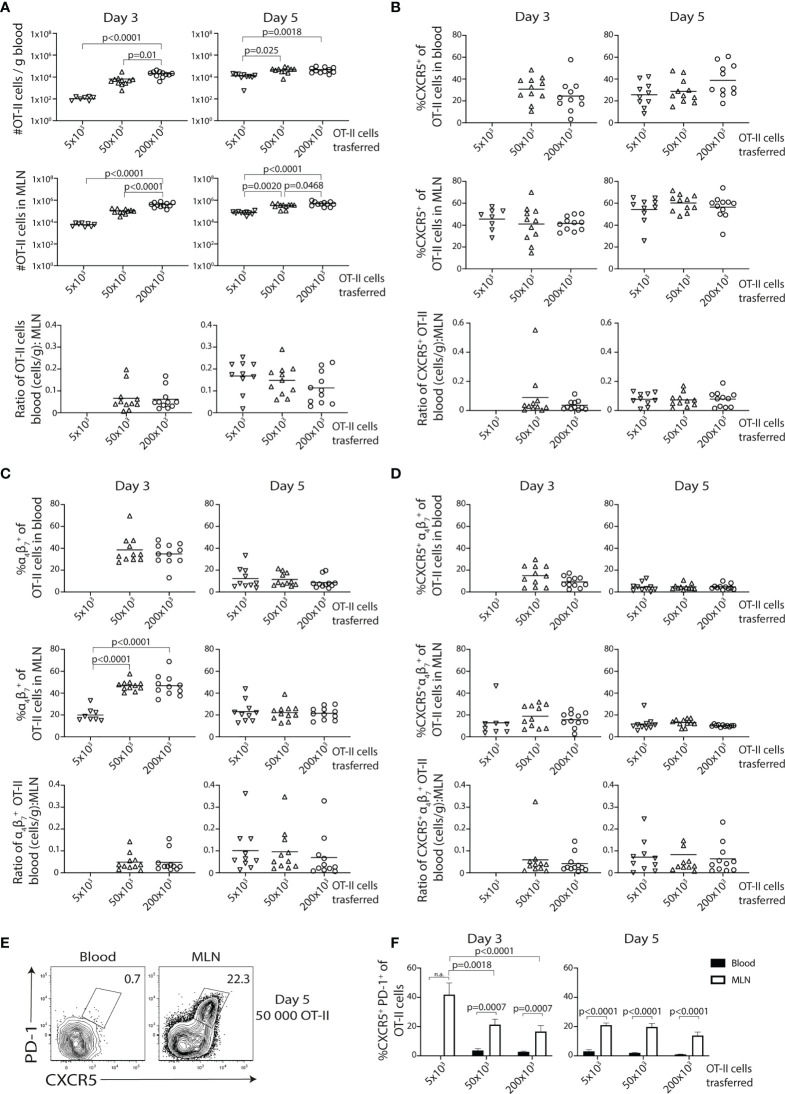
Increasing numbers of precursor cells promote the initial generation of α_4_β_7_^+^ cells but does not impair the development of CXCR5-expressing cells. **(A–F)** Titrated numbers of CD45.1^+^ OT-II cells were adoptively transferred and then analyzed in mesenteric lymph node (MLN) and blood at indicated time points post-immunization with OVA + polyI:C. **(A)** Graphs showing the total number of OT-II cells per gram of blood, in the MLN and the relative egress (ratio OT-II cells in blood:MLN) for each precursor number at indicated time points post-immunization. Bar graphs showing the frequency and relative egress of CXCR5^+^ **(B)**, α_4_β_7_^+^ **(C)**, and CXCR5^+^ α_4_β_7_^+^ **(D)** OT-II cells in blood and MLN. **(E)** Contour plots showing CXCR5 and PD-1 expression by OT-II cells in blood and MLN at 5 days post-immunization using 50,000 OT-II precursor cells and **(F)** a bar graph showing the frequency of CXCR5^+^PD-1^+^ of OT-II cells in blood (black bars) and MLN (white bars) 3 and 5 days post-immunization using different numbers of adoptively transferred cells. Pooled data from three independent experiments, *n* = 8–11per group. One-way ANOVA with Tukey’s multiple comparison test; *p*-values < 0.05 are reported.

To assess if the relatively high numbers of transferred OT-II cells affect the generation and egress of the CXCR5^−^ α_4_β_7_^+^ effector Th and CXCR5^+^PD-1^+^ Tfh-like cell subsets, we titrated the number of transferred cells. To reduce the technically demanding cannulations of the thoracic duct, terminal peripheral blood was collected at different time points following immunization. The transfer of 5 × 10^3^ OT-II cells resulted in too few OT-II cells in the circulation (100 cells/g blood) for phenotypic analyses 3 days post-immunization (**Figures 2A–D**). Analysis at day 5 revealed no pronounced differences in the number of OT-II cells recovered from the blood or MLN after transfer of 50 or 200 × 10^3^ cells, while significantly fewer OT-II cells were found in both organs when using 5 × 10^3^ cells (**Figure 2A**). The frequency of CXCR5^+^ OT-II cells in the blood or MLN was however consistently unaffected by the number of transferred cells (**Figure 2B**). In contrast, the frequency of α_4_β_7_ expressing OT-II cells was increased in the MLN at day 3 when comparing the lowest cell transfer number with 50 or 200 × 10^3^ cells (**Figure 2C**), suggesting that the pronounced output of α_4_β_7_^+^ OT-II cells into the lymph 3 days after immunization (see **Figures 1G, H** **)** at least partially is related to the higher number of transferred OT-II cells in these experiments. However, by day 5, the frequencies of α_4_β_7_^+^ OT-II cells were normalized among the groups (**Figure 2C**), indicating that this effect of transferring a higher number of OT-II cell was only transient. Furthermore, when focusing on the CXCR5^+^α_4_β_7_^+^ subset, we observed no changes in frequencies when more OT-II cells were transferred (**Figure 2D**). While the transfer of 50 and 200 × 10^3^ cells numerically rendered a greater overall recovery of cells expressing α_4_β_7_ and/or CXCR5 (**Supplementary Figures 2A–C**), no difference was observed in the ratio of the number of cells in the blood to MLN (reflecting egress efficiency), irrespective of the phenotype being assessed (**Figures 2A–D**). Finally, no CXCR5^+^PD-1^+^ T cells could be detected in the blood (**Figures 2E, F** **)**, confirming previous results (16). In contrast, this population was readily detected in the MLN (**Figures 2E, F** **)** and lymph (**Figures 1E, F** **)** 5 days after immunization.

Collectively, these titration experiments show that higher precursor numbers are required for phenotypic studies of circulating OT-II cells at day 3. While the higher number of transferred cells leads to an initially biased generation of α_4_β_7_^+^ gut-homing effector cells that is normalized by day 5, it does not influence the expression of CXCR5 or the efficiency by which the OT-II cells exit the LN.

### The Appearance of Tfh-Like Cells in the Efferent Lymph Requires Cognate B-Cell Interactions

**Figure 3:**
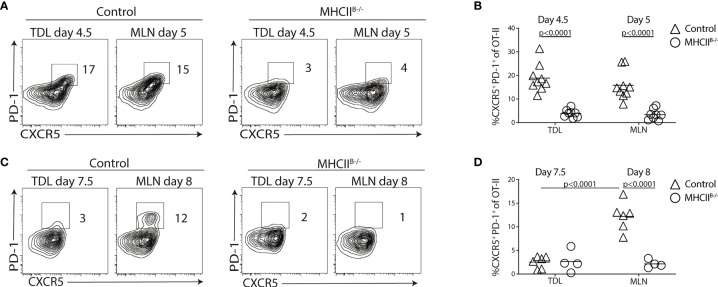
Cognate T-B cell interactions are required for generation of CXCR5^+^PD-1^+^ TDLs. **(A–D)** CD45.1^+^ OT-II cells were adoptively transferred into control or MHCII^B-/-^ chimeras that were immunized with OVA + polyI:C the following day. TDLs were collected at indicated intervals prior to sacrifice and harvest of MLN 5 or 8 days post-immunization for parallel flow cytometry analyses. **(A–D)** Representative flow cytometry analysis of CXCR5 and PD-1 among OT-II TDLs and in MLNs **(A)** and frequency of CXCR5^+^ PD-1^+^ of OT-II cells in each group **(B)** at 5 days post-immunization or 8 days post-immunization **(C, D)**. **(A, B)** represent two individual experiments, n=7-9 and **(C, D)** two individual experiments, n=4-6. One-way ANOVA with Sidak’s multiple comparison test was used for statistical analysis, p-values <0.05 are reported.

We next asked the question whether the appearance of Tfh-like cells in the lymph was dependent on cognate B-cell interactions. For this, we conducted cannulation experiments using MHCII^B−/−^ bone marrow chimeras transferred with 2 × 10^5^ OT-II cells. Similar frequencies of total and α_4_β_7_^+^ Tfh-like OT-II cells were detected in the TDLs and MLNs of control chimeras 5 days after immunization, indicating an efficient egress of Tfh-like cells from the LNs at this time point (**Figures 3A, B** and **Supplementary Figures 3A, B** **)**. In contrast, CXCR5^+^PD-1^+^ OT-II cells were not present in TDL or MLN of MHCII^B−/−^ mice (**Figures 3A, B** and **Supplementary Figures 3A, B** **)**. Analysis 8 days after immunization revealed that a distinct subset of CXCR5^+^PD-1^bight^ GC-Tfh cell was present in the MLNs of control but not in MHCII^B−/−^ chimeras (**Figure 3C**). This is in agreement with previous studies, demonstrating an essential role for B cells in the generation of *bona fide* Tfh cells (34–38). Still, at this later time point, essentially no CXCR5^+^PD-1^+^ OT-II cells could be detected in the efferent lymph, regardless of whether TDLs from MHCII^B−/−^ or wild-type chimeras were analyzed (**Figures 3C, D** and **Supplementary Figures 3C, D** **)**. These results show that cognate B-cell interactions are required for the generation of Tfh-like cells in the MLN and for their subsequent appearance in the efferent lymph. Furthermore, the limited time period when these cells can be found in the lymph (after day 3 but before day 8 after immunization) precedes the appearance of *bona fide* CXCR5^+^PD-1^bright^ GC-Tfh cells in the MLNs.

### Loss of Tfh-Like Cells in the Absence of MHC-II on B Cells Is Accompanied by a Transiently Increased Proportion of α_4_β_7_^+^ T Cells in the Efferent Lymph

**Figure 4:**
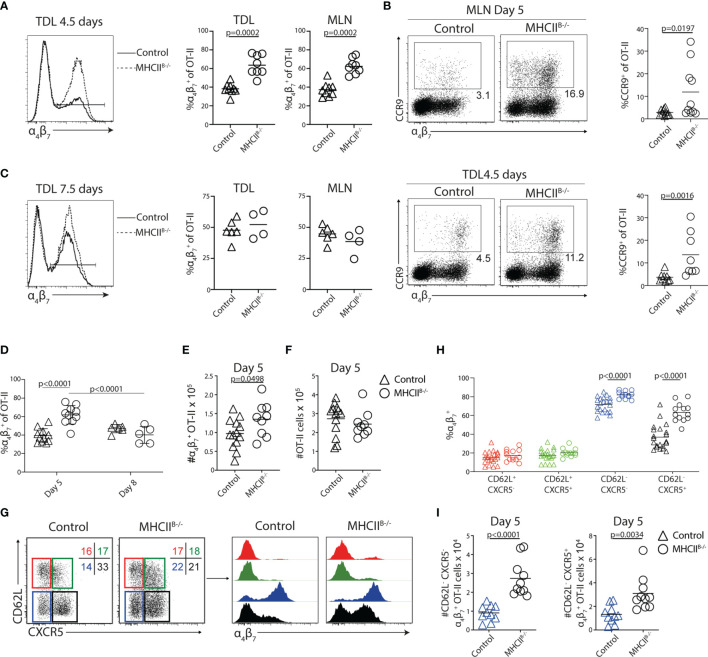
Lack of cognate T–B-cell interactions causes a transient increase of α_4_β_7_^+^ OT-II cells in the efferent lymph and MLN. **(A–I)** CD45.1^+^ OT-II cells were adoptively transferred into control or MHCII^B−/−^ chimeras that were immunized with OVA + poly I:C the following day. TDLs were collected **(A–C)** at indicated intervals prior to sacrifice and harvest of MLN 5 or 8 days post-immunization for parallel flow cytometry analyses. **(A)** Expression of α_4_β_7_ by OT-II TDLs from control and MHCII^B−/−^ and frequency of α_4_β_7_^+^ cells among OT-II cells collected in the lymph or MLN from pooled experiments 4.5/5 days post-immunization. **(B)** Expression of CCR9 and α_4_β_7_ by OT-II from the MLN (upper) and efferent lymph (lower) in control or MHCII^B−/−^ chimeras and frequency of CCR9^+^ cells among OT-II cells collected in the MLN or lymph from pooled experiments. **(C)** α_4_β_7_ expression by OT-II cells and frequency of α_4_β_7_^+^ of OT-II cells in the efferent lymph and MLNs of control and MHCII^B−/−^ mice 7.5/8 days post-immunization. **(D)** Frequency of OT-II cells in the MLNs expressing α_4_β_7_ in control and MHCII^B−/−^ mice from pooled experiments. **(E, F)** Number of **(E)** α_4_β_7_^+^ OT-II cells and **(F)** OT-II cells in the MLNs 5 days post-immunization. **(G)** Expression of CD62L and CXCR5 by OT-II cells 5 days post-immunization and gates used to identify four subpopulations for further analyses of α_4_β_7_ expression. **(H)** Frequency of α_4_β_7_^+^ cells within each subpopulation and **(I)** number of α_4_β_7_^+^ cells within the CD62L^−^CXCR5^−^ (left) and CD62L^−^CXCR5^+^ (right) populations. **(A, C)** Two individual experiments, *n* = 7–9 and **(B)** two individual experiments, *n* = 4–6. **(D)** Pooled data from two independent experiments, *n* = 5–11 and **(E–I)** data from three independent experiments, *n* = 7–13. Two-tailed unpaired Student’s *t*-test of one-way ANOVA with Sidak’s multiple comparison test was used for statistical analysis; *p*-values < 0.05 are reported.

While α_4_β_7_^+^ Tfh-like cells were readily detected in the efferent lymph of wt mice 5 days p.i., the integrin was not expressed by GC-Tfh cells in the MLN of these mice 8 days after immunization (**Supplementary Figures 3C, D** **)**. Given that the GC-Tfh cell phenotype depends on sustained cognate B-cell interaction (39), the results suggested that a prolonged interaction with B cells may prevent α_4_β_7_ expression by the GC-Tfh cell subset. We therefore sought to determine if and to what extent antigen-presenting B cells have an impact on the α_4_β_7_ *^+^* T cells that enter the efferent lymph after immunization. A higher proportion of the TDL OT-II cells expressed α_4_β_7_ in MHCII^B−/−^ recipients (64% ± 11%; mean value ± SD) compared with controls (38% ± 6%; mean value ± SD) 5 days post-immunization (**Figure 4A**). This was also observed in the MLN (**Figure 4A**). The proportion of CCR9^+^ cells was also increased in TDL and MLN of MHCII^B−/−^ recipients (**Figure 4B**). At the day 8 collection, the frequency of α_4_β_7_ *^+^* OT-II cells had however normalized in MHCII^B−/−^ recipients (**Figure 4C**). To determine if the observed increase in frequencies of α_4_β_7_ *^+^* OT-II cells was caused by an increase in absolute number of this subset and to avoid any potential influence that collection of lymph might have on the recovery of cells from the MLN, non-cannulated control and MHCII^B−/−^ recipients were sacrificed at days 5 and 8 post-immunization. Consistent with the results obtained for the TDL preparations, the frequency and number of α_4_β_7_^+^ OT-II cells, but not total OT-II cells, were increased in MHCII^B−/−^ chimeras 5 days after immunization (**Figures 4D–F**). Also similar to the efferent lymph, this difference between MHCII^B−/−^ and control mice could not be seen in the MLN 8 days after immunization. Antigen-presenting B cells therefore appear to actively counteract the generation of gut-homing T cells in the MLN 5 days p.i. and thereby their appearance in the efferent lymph.

Teff cells with tropism for peripheral tissues, including α_4_β_7_^+^ gut-homing T cells, can be distinguished by their lack of CD62L (20). Likewise, Tfh cells are also CD62L^−^ (40). This is in contrast to CD62L^+^ central memory Th cells, which in general do not access parenchymal tissues or GCs but rather retain the blood to LN recirculation pattern of naive T cells (41, 42). We therefore next addressed if the ability of B cells to supress α_4_β_7_ expression, as observed 5 days p.i., mostly affects Th cells with central memory characteristics, the CD62L^−^ subset that normally targets peripheral tissues and/or the Tfh cell lineage. Analysis of MLNs from control bone marrow chimeras 5 days after immunization revealed that α_4_β_7_ was predominantly found on CD62L^−^ OT-II cells and, in particular, on the CXCR5^−^ subset (**Figures 4G, H** **)**. In MHCII^B−/−^ mice, CD62L^+^ OT-II cells remained negative for α_4_β_7_. The proportion and number of α_4_β_7_^+^ OT-II cells were instead significantly increased both among CD62L^−^CXCR5^−^ cells and, in particular, within the CD62L^−^CXCR5^+^ subset (**Figures 4H, I** **)**.

We extended our experiments to include also μMT mice and CD19-Cre × MHCII^fl/fl^ mice, representing a model completely devoid of B cells and an alternative model with MHC-II loss confined to the B-cell lineage, respectively. In both these models, the proportional increase in Th cells expressing α_4_β_7_ was most pronounced for the CD62L^−^CXCR5^+^ subset (**Supplementary Figures 4A, B** **)**. Moreover, OT-II cells primed in control and MHCII^B−/−^ mice in response to orally administered OVA and cholera toxin (CT) expanded equally, with increased expression of α_4_β_7_ in the absence of cognate B-cell interactions (**Supplementary Figures 5A, B** **)**. Again, this appeared more pronounced for the CD62L^−^CXCR5^+^ subset (**Supplementary Figures 5C, D** **)**. Hence, B cells reduce the frequencies of α_4_β_7_^+^ cells among the CD62L^−^ Th cell subsets.

### Generation of IL-4-Producing Th Cells Requires Antigen-Presenting B Cells

**Figure 5:**
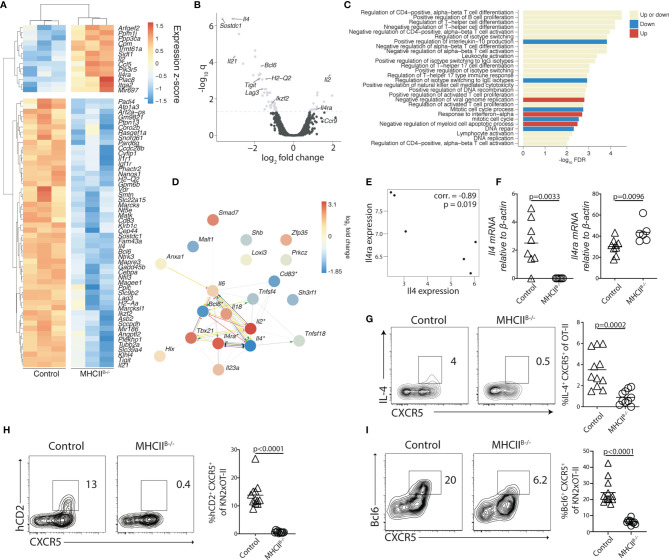
B cells regulate the expression of IL-4 and its receptor IL4Rα in Tfh cells. **(A–G)** Adoptively transferred OT-II **(A–G)** or **(H, I)** KN2xOT-II cells from control or MHCII^B−/−^ chimeras were analyzed 5 days post-immunization with OVA and polyI:C. **(A)** Differentially expressed genes (*q* < 0.05) of sorted total OT-II cells from the MLN between the MHCII^B−/−^ and control conditions (*n* = 3 per group). Colors indicate *z*-scores of RMA-normalized gene expression values. Genes and samples were hierarchically clustered with a Euclidean distance metric and complete linkage. **(B)** Volcano plot showing differentially expressed genes (*q* < 0.05 above the dashed line) and their relative fold change. **(C)** Enriched Gene Ontology biological processes (*q* < 0.05) among the list of all genes ranked by their fold changes, determined with the STRING database gene set enrichment function. Bars are colored by whether the respective categories were enriched at the top of the ranked gene list (upregulated), the bottom (downregulated), or at both ends (both up- and downregulated genes). **(D)** Genes in the most significantly enriched category visualized as a network with STRING. Colors of nodes indicate log_2_ fold changes. Asterisks indicate differential expression at *q <* 0.05. Lines indicate evidence of interaction from the sources “Textmining,” “Experiments,” or “Databases” at the default confidence threshold of 0.4. Colors of lines indicate the type of action: binding (blue), inhibition (red), transcriptional regulation (yellow), reaction (black), catalysis (purple), or unspecified (gray). Arrows at the end of the lines indicate positive action, and perpendicular bars indicate negative action. **(E)** Relationship between the expression of *Il4* and *Il4ra* (“corr.”: Pearson correlation coefficient). **(A–E)** Data from one experiment, *n* = 3 per group. **(F)** qRT-PCR analysis of expression levels of *Il4* and *Il4ra* relative to *Actb* in OT-II cells sorted from the MLNs of control and MHCII^B−/−^ mice, 2 individual experiments in which *n* = 9/6. **(G)** Splenocytes from control and MHCII^B−/−^ were restimulated with PMA and ionomycin for 4 h in the presence of brefeldin **(A)** Flow cytometry plots show intracellular staining for IL-4 with data summarized in bar graphs, data from two individual experiment, *n* = 10–11. **(H, I)** KN2xOT-II cells adoptively transferred into control or MHCII^B−/−^ and MLNs were analyzed 5 days p.i. for hCD2 **(H)** and Bcl6 **(I)** expression, data from two individual experiments where *n* = 12/10. Statistical analysis was performed using unpaired Student’s *t*-test; *p*-values < 0.05 are reported.

To gain insight into the transcriptional program conferred to Th cells by B cells, we next conducted microarray gene expression analyses of OT-II cells sorted from the MLN of control and MHCII^B−/−^ mice 5 days after immunization. A total of 65 genes were found to be differentially expressed (*q* < 0.05, 48 over 2-fold) in the absence of cognate B–T-cell interaction (**Figures 5A, B** **)**. Consistent with the established role for B cells in the final Tfh cell fate commitment, a number of Tfh cell-associated genes, including *Bcl6*, *Lag3*, *Ikzf2*, *Tigit*, and *Il21*, were downregulated in the absence of MHC-II on B cells (**Figures 5A, B** **)**. Reversely, the expression of *Il2*, which is known to antagonize Tfh cell development through induction of Blimp-1 (43, 44), was increased in the absence of antigen presentation by B cells. Further analysis of regulated gene sets revealed that genes associated with conventional CD4^+^ T-cell differentiation constituted one of the most affected categories of genes (**Figures 5C, D** **)**. Protein–protein interaction network analysis of the aforementioned category identified *Tbx21*, *Il2*, *Bcl6*, *Il4*, and *Il4ra* as interaction partners with significantly regulated gene expression (**Figure 5D**). Focusing on the genes with significantly reduced expression in MHCII^B−/−^ recipient mice, we found that *Il4* was among the most affected, with an almost 9-fold decrease in the absence of MHC-II expressing B cells. The reduced amounts of *Il4* mRNA correlated with increased expression of the IL-4 receptor subunit *Il4ra* (**Figure 5E**). We confirmed these results by qRT-PCR analysis, which revealed that while *Il4* expression was readily detected in OT-II cells sorted from control recipients, it was reduced below the detection limit in all MHCII^B−/−^ recipients (**Figure 5F**). We also confirmed a significantly higher expression of *Il4Rα* when cognate B-cell interaction was prevented (**Figure 5F**). To examine if the absence of *Il4* mRNA in CD4 T cells from MHCII^B−/−^ mice was due to differentiation events, or rather reflected the lack of continuous TCR signaling (45), we restimulated OT-II cells from bone marrow chimeras 5 days after immunization. After a brief *ex-vivo* stimulation with PMA/ionomycin, cells from control chimeras produced IL-4 that was completely confined to the CXCR5^+^ subset (**Figure 5G**). In contrast, the stimulation failed to induce IL-4 production from OT-II cells primed in MHCII^B−/−^ mice (**Figure 5G**).

Low-level production of IL-4 is difficult to detect through intracellular staining with antibodies but can be visualized in KN2/KN2 mice where transgenic expression of human CD2 serves as a retained membrane-anchored reporter for IL-4 secretion (24). We therefore crossed OT-II mice with the KN2/KN2 strain and transferred KN2xOT-II cells into MHCII^B−/−^ and control chimeras. As CD45.2 could not be used as a congenic marker in these experiments, donor cells were instead detected based on Vα2 and Vβ5.1 TCR expression. To exclude a minor population of recipient-derived Vα2 and Vβ5.1 expressing cells, we took advantage of the selective increase in PD-1 expression on antigen-activated T cells (**Supplementary Figure 1E**). Five days after immunization, corresponding to the time point when LN egress peaks, approximately 13% of all MLN KN2xOT-II cells from control chimeras expressed detectable amounts of the transgenic IL-4 reporter, with expression confined to cells co-expressing high levels of CXCR5 (**Figure 5H**). By contrast, no expression of hCD2 could be observed in the MHCII^B−/−^ recipients (**Figure 5H**).

The transcriptional repressor Bcl6 is required for the Tfh cell differentiation program and, perhaps, serves as the most reliable marker for mature Tfh cells in lymphoid tissues (3). However, cognate interaction with B cells is dispensable for the initial upregulation of Bcl6 in developing Tfh cells (34, 46). Consistent with this, we found Bcl6 to be less affected than transgenic hCD2 expression in the MHCII^B−/−^ recipient mice, although the transcriptional repressor also was significantly reduced in these mice (**Figure 5I**).

Taken together, these results show that antigen-presenting B cells are required for the generation of IL-4-producing Th cells in the poly I:C-driven immune response. Furthermore, they indicate that transgenic hCD2 expression on KN2xOT-II cells can serve as a retrospective marker for Th cells that have undergone cognate interactions with B cells.

### Production of IL-4 by Developing Tfh Cells Occurs Before Entry Into GCs and Only in Close Proximity to B Cells

**Figure 6:**
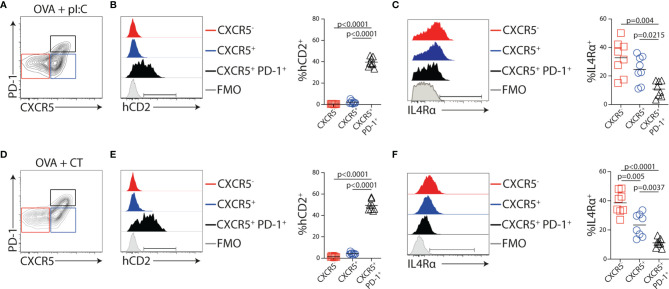
IL-4 secretion and IL4Rα downregulation in Tfh cells are not dependent on the adjuvant used. **(A–F)** KN2xOT-II cells were adoptively transferred into wt recipients that received OVA and polyI:C **(A–C)** or OVA and CT **(D–F)** i.p. the following day. MLNs were collected 5 days post-immunization for flow cytometry analyses. **(A, D)** Contour plots showing gates used to identify CXCR5^−^, CXCR5^+^, and CXCR5^+^PD-1^+^ KN2x-OT-II populations. **(B, C, E, F)** Expression of **(B, E)** hCD2 and **(C, F)** IL4Rα following immunization with the indicated adjuvant by the gated populations and frequency of the indicated populations from pooled experiments. Data from two individual experiments where *n* = 8 for each adjuvant. Statistical analysis was performed using one-way ANOVA with Tukey’s multiple comparison test; *p*-values < 0.05 are reported.

**Figure 7:**
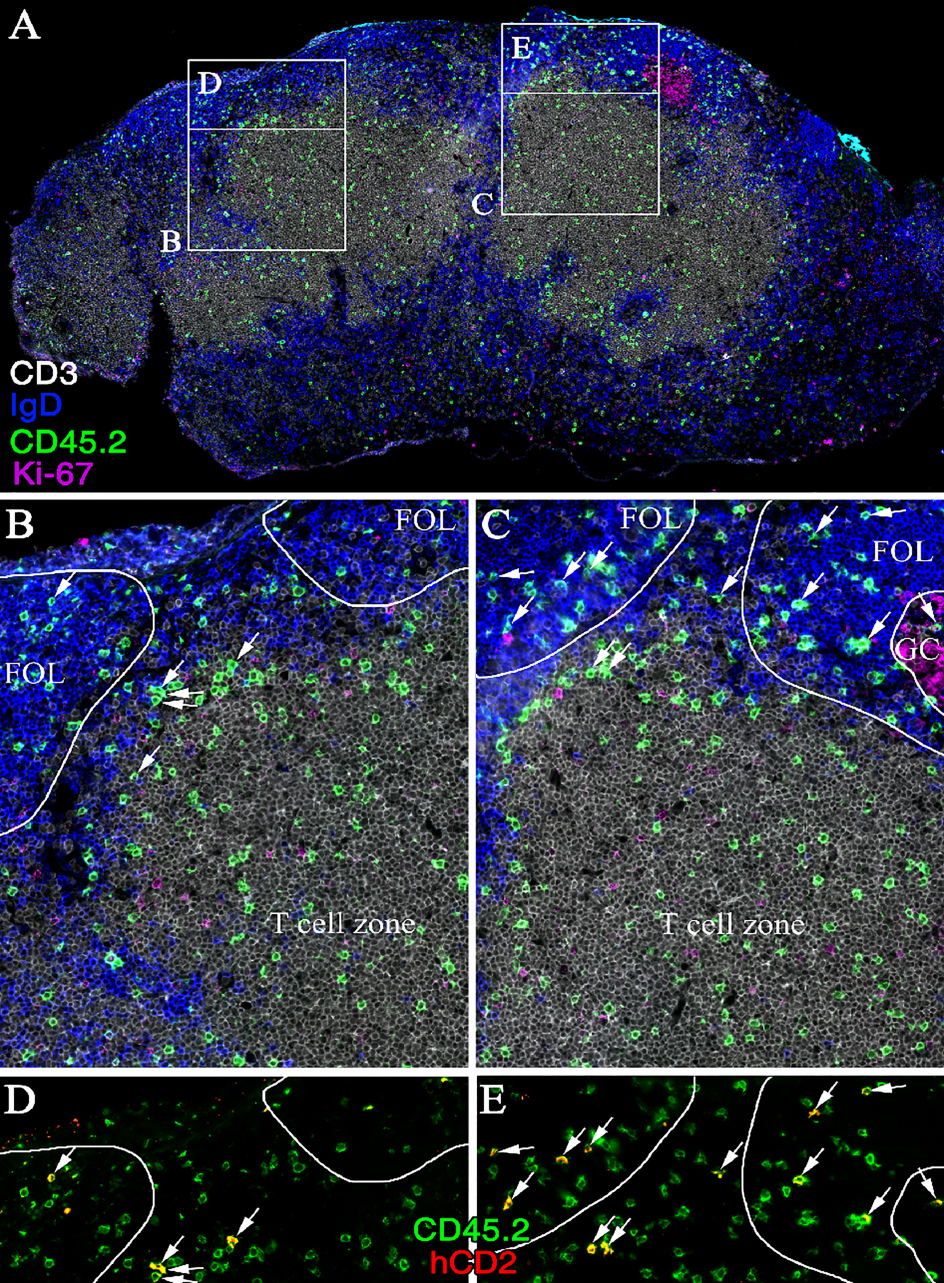
IL-4 secreting Th cells are localized to the interfollicular region and B-cell follicle following polyI:C immunization. Immunofluorescence staining of **(A)** a cross-section of cryopreserved MLN 5 days post-immunization of a CD45.1 mouse receiving KN2xOT-II transfer. Zoom-ins of T-cell zone and B-cell follicles **(C)** with or **(B)** without a forming GC as well as **(D, E)** close-ups of respective interfollicular region are shown. Sections were stained for CD3 (white), IgD (blue), CD45.2 (green), Ki-67 (magenta), and hCD2 (red); arrows indicate cells expressing CD45.2 and hCD2; image representative of six stained MLNs.

IL-4 production in reactive LNs was largely tracked with Tfh cells during Th2-biased immune responses (47). To assess the identity of the IL-4-producing cells after OVA + poly I:C immunization, KN2xOT-II cells were transferred to CD45 congenic wild-type mice. Five days post-immunization, we analyzed MLNs from recipients by flow cytometry. At this time point, the expression of hCD2 was restricted to CXCR5^+^PD-1^+^ Tfh cells (**Figures 6A, B** **)**. In contrast, the surface expression of IL4Rα was reduced on these cells as compared with the CXCR5^−^ or CXCR5^+^PD-1^−^ subsets (**Figure 6C**). Similar Tfh-restricted expression of hCD2 was observed when recipients were immunized with the Th1/Th2/Th17-inducing adjuvant CT (**Figures 6D, E** **)**. Notably, the frequency of hCD2^+^ OT-II cells only differed slightly between mice immunized with poly I:C and CT, indicating that the choice of adjuvant has little or no impact on the ability of Tfh cells to produce IL-4 (**Figure 6F**). While CXCR5^+^PD-1^+^ Tfh cells are mostly localized to GCs 8 days after immunization (36) and, at this time point, fail to enter efferent lymph (see **Figures 1E**, **3B**), we next tracked the localization of IL-4-producing cells in the MLN at the peak of their exit into efferent lymph. For this, we performed immunofluorescence analysis of the recipient mice 5 days after immunization. KN2xOT-II cells expressing hCD2 were identified in the interfollicular regions, at the T/B-cell border, and in the T zone-proximal area of the B-cell follicles (**Figure 7**). While small clusters of Ki67^+^ proliferating B cells could be observed in some B-cell follicles, IL-4-producing KN2xOT-II cells primarily localized to B-cell follicles regardless of whether they contained Ki67^+^ GCs or not (**Figure 7**). Very few if any hCD2^+^ KN2xOT-II cells could be identified in the T-cell zone. These results therefore show that developing Tfh cells acquire the ability to produce IL-4 before they take up residency within established GCs and that this appears to primarily occur in close proximity to B cells.

### Tfh-Like Cells Associated With Past and/or Present IL-4 Production Enter the Efferent Lymph Following Immunization

**Figure 8:**
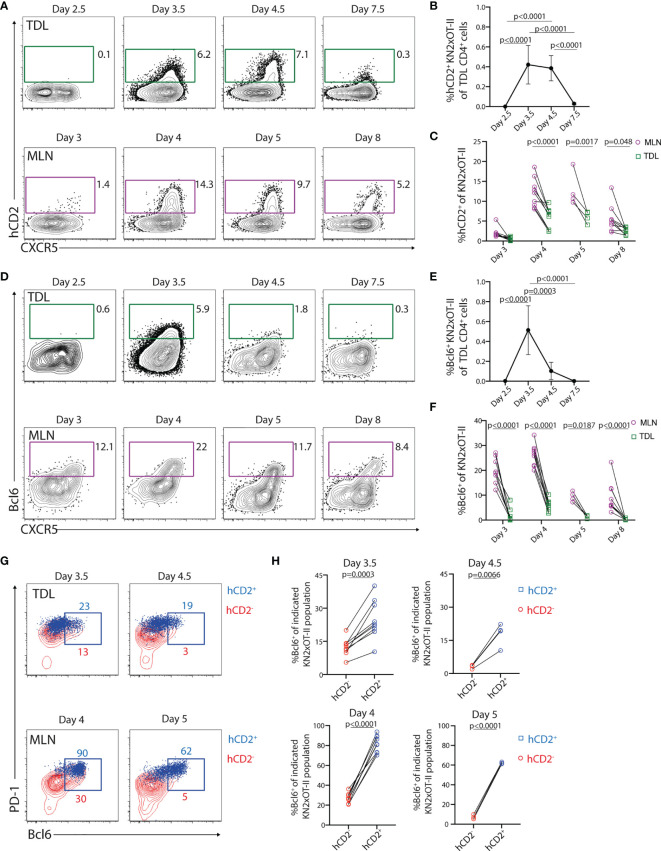
Tfh-like cells with a history of IL-4 secretion are present in the efferent lymph during a limited period of time. **(A–H)** KN2xOT-II cells were adoptively transferred into wild-type mice that received OVA and polyI:C. TDLs were collected at indicated intervals prior to sacrifice and harvest of MLN 3, 4, 5, and 8 days post-immunization for parallel flow cytometry analyses. **(A)** hCD2 and CXCR5 expression by KN2xOT-II cells in the efferent lymph and MLNs. **(B)** Proportion of hCD2^+^ KN2xOT-II cells among CD4^+^ TDLs. **(C)** Frequency of hCD2^+^ among KN2xOT-II cells in the MLN and efferent lymph from pooled experiments. **(D)** Expression of Bcl6 and CXCR5 by KN2xOT-II cells in the efferent lymph and MLNs. **(E)** Proportion of Bcl6^+^ KN2xOT-II cells among CD4^+^ TDLs. **(F)** Frequency of Bcl6^+^ cells among KN2xOT-II cells in the MLN and efferent lymph from pooled experiments. **(G)** Expression of PD-1 and Bcl6 by hCD2^+^ (blue dot plots) and hCD2^−^ (red contour plots) KN2xOT-II cells in TDL (upper) and MLNs (lower) at indicated times. **(H)** Frequency of Bcl6^+^ cells of hCD2^+^ (blue squares) and hCD2^−^ (red circles) KN2xOT-II cells in TDL (upper) and MLN (lower) at indicated time points. Data from two individual experiments with the exception for day 4.5/5 which was performed once, *n* = 4–9. Statistical analysis was performed using two-tailed paired Student’s *t*-test **(H)** or one-way ANOVA with Tukey’s **(B, E)** or Sidak’s **(C, F)** post-test; *p*-values < 0.05 are reported.

We next wished to assess if transgenically marked KN2xOT-II cells enter the efferent lymph. For this, we collected TDLs on days 3, 4, 5, and 8 after immunization (i.e., 48–72, 72–96, 96–120, and 168–192 h post-immunization). KN2xOT-II cells with detectable hCD2 expression were absent in the first collection but appeared among the CXCR5^+^ subset day 4 after immunization and remained at a similar frequency during day 5 (**Figure 8A**). Thereafter, hCD2^+^ cells declined in the lymph to be essentially absent from TDLs 8 days after immunization. This was particularly clear when assessing the percentage of hCD2^+^ KN2xOT-II cells among total efferent lymph CD4^+^ T cells (**Figure 8B**). This kinetics mirrored the appearance of hCD2^+^ cells in the MLN (**Figure 8A**). The percentage of KN2xOT-II cells expressing hCD2 was however consistently higher in the MLN than in the TDL preparations. This indicated a decline in the expression level of the hCD2 transgene after LN egress and/or that IL-4-producing cells are relatively efficiently retained in the LN (**Figure 8C**). Taken together, these results demonstrate that Tfh-like cells with past and/or present IL-4 production enter the efferent lymph, a process that can be traced in the KN2xOT-II adoptive cell transfer model through retained transgenic expression of hCD2.

### IL-4-Producing Tfh-Like TDLs Originate From the Bcl6^+^ Tfh Cell Lineage in Lymph Nodes But Rapidly Lose Expression of Bcl6

Detecting Bcl6 expression in circulating Tfh-like cells has been difficult (16, 48). This could potentially reflect that Bcl6 expression is lost from Tfh-like cells during their transition from the LN to blood. Alternatively, Bcl6-expressing Tfh cells might be retained in the LNs and, thus, were distinct from the IL-4-producing CXCR5^+^PD-1^+^ Tfh-like cells that enter efferent lymph. To assess this, we analyzed Bcl6 expression in conjunction with hCD2 on the KN2xOT-II cells the in efferent lymph and MLN. Among the TDLs, Bcl6 expression was clearly detected at 72–96 h p.i. but peaked with more rapid kinetics as compared with hCD2 (**Figures 8B, D, E** **)**. In line with the reduced expression of hCD2 in TDL compared with MLN (**Figure 8C**), Bcl6 expression was consistently lower in KN2xOT-II cells in TDL compared with MLN (**Figures 8D, F** **)**. Essentially, all hCD2^+^ KN2xOT-II cells in the MLN expressed Bcl6, demonstrating a link between the Tfh cell lineage and IL-4 production in the MLN (**Figures 8G, H**, lower row). Consistent with this, a significantly higher proportion of hCD2^+^ than hCD2^−^ KN2xOT-II cells in the lymph expressed Bcl6 (**Figures 8G, H**, upper rows). Thus, even though very few Bcl6^+^ cells enter the lymph (**Figure 8E**), the ones that do enter are enriched in the KN2xOT-II cell subset with detectable hCD2 expression (i.e., current and/or past IL-4 producers). Collectively, these results indicate that the exiting IL-4-producing Tfh-like cells originate from a Bcl6^+^ intermediate differentiation stage in the LNs and that the majority of these cells lose expression of Bcl6 already 4–5 days after immunization, regardless of whether they remain or leave the LNs.

### Tfh-Like Cells Enter the SI-LP

**Figure 9:**
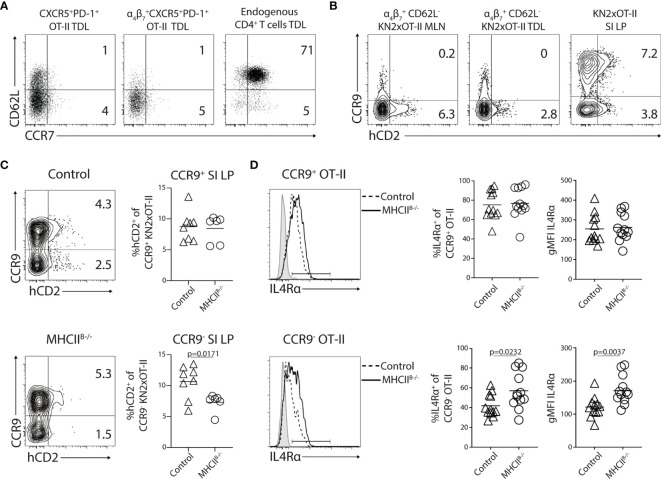
CCR9^−^CXCR5^+^PD-1^+^ Th cells enter small intestinal lamina propria (SI-LP). **(A–D)** KN2xOT-II cells were adoptively transferred to wild type **(A, B)** or control and MHCII^B−/−^ chimeras **(C, D)** that received OVA + polyI:C i.p. the following day. MLNs, SI-LP were harvested 5 days post-immunization and TDLs were collected 4.5 days p.i. for flow cytometry analyses. **(A)** Expression of CD62L and CCR7 by CXCR5^+^PD-1^+^ or α_4_β_7_^+^ CXCR5^+^PD-1^+^ KN2xOT-II TDLs and endogenous CD4^+^ TDLs. **(B)** Expression of CCR9 and hCD2 by α_4_β_7_^+^ CD62L^−^ cells in the MLN, efferent lymph, and total KN2xOT-II in SI-LP. **(C)** Expression of CCR9 and hCD2 by SI-LP KN2xOT-II cells in control and MHCII^B−/−^ chimeras. Frequency of hCD2^+^ among CCR9^+^ and CCR9^−^ KN2xOT-II cells pooled from two independent experiments, *n* = 8/6. **(D)** Expression of IL4Rα by CCR9^+^ (upper) and CCR9^−^ (lower) SI-LP KN2xOT-II cells in control or MHCII^B−/−^ chimeras. Frequency of IL4Rα^+^ cells (left) and IL4Rα gMFI (right) among CCR9^+^ and CCR9^−^ SI-LP KN2xOT-II cells pooled from four independent experiments, *n* = 17/14. Unpaired two-tailed Student’s *t*-test was used for statistical testing; *p*-values < 0.05 are reported.

Circulatory Tfh-like cells are currently thought to primarily serve as a reservoir of memory Th cells with an enhanced ability to provide help in secondary antibody responses in secondary lymphoid organs (16, 48, 49). Assessment of Tfh-like OT-II TDLs day 5 after immunization revealed that the vast majority were negative for CD62L and CCR7 (**Figure 9A**). This extended to the α_4_β_7_^+^ Tfh-like TDLs (**Figure 9A**). Therefore, we next explored the possibility that Tfh-like cells that have undergone cognate B-cell interaction can enter peripheral tissues. For this, we adoptively transferred KN2xOT-II cells into MHCII^B−/−^ or control recipient mice. Thereafter, we prepared single-cell suspensions of SI-LP 5 days after immunization. While CXCR5 and PD-1 were poorly preserved after the enzymatic digestion and, therefore, could not be analyzed on SI-LP cells, hCD2 expression could be detected on SI-LP KN2xOT-II cells. The transgenically marked donor cells were present at similar frequency in the intestine of MHCII^B−/−^ and control recipient mice (**Supplementary Figure 6A**). Also, analysis of the frequency of total OT-II cells among leukocytes in SI 5 days after immunization revealed no difference between MHCII^B−/−^ and control chimeras (**Supplementary Figure 6B**).

We next reasoned that Tfh-like cells that possibly enter the SI-LP might be difficult to distinguish due to the large cohort of conventional gut-homing T cells entering this site earlier in the response. To exclude conventional α_4_β_7_^+^CCR9^+^ donor cells from our SI-LP analysis, we decided to analyze hCD2 in conjunction with CCR9 expression. We first wanted to confirm that the few CCR9^+^ Th cells that exit the MLN later in the response are distinct from the IL-4-producing subset. For this, we first collected the MLNs, TDLs, and SI-LP 5 days p.i. from wt mice adoptively transferred with KN2xOT-II cells. In the MLNs and TDL, the expression of CCR9 and hCD2 by CD62L^−^ α_4_β_7_^+^ KN2xOT-II was mutually exclusive (**Figure 9B**). In contrast, hCD2 could be detected on both the CCR9^+^ and CCR9^–^ subset (**Figure 9B**) and to a similar extent (**Supplementary Figure 6C**) in the SI-LP, indicating that the CCR9^+^ subset becomes hCD2^+^ after entering the intestinal mucosa. Therefore, we next assessed the SI-LP CCR9^+^ and CCR9^−^ KN2xOT-II cell subsets separately in control versus MHCII^B−/−^ chimeras. While the frequency of hCD2^+^ was similar among CCR9^+^ donor cells in the two groups of recipient mice, it was significantly reduced among CCR9^−^ OT-II cell in MHCII^B−/−^ mice (**Figure 9C**). IL4Rα expression is downmodulated on CXCR5^+^PD-1^+^ cells in the MLN (**Figure 6C**) and Tfh-like cells in the lymph (**Supplementary Figures 6D, E** **)**. Therefore, as a second readout for previous interactions with B cells, we assessed B-cell-dependent downmodulation of IL4Rα. This revealed that CCR9^−^, but not CCR9^+^ KN2xOT-II SI-LP cells, presented with increased expression of IL4Rα in MHCII^B−/−^ chimeras (**Figure 9D**). Altogether, these experiments indicate that Tfh-like cells entering the circulation after priming in the MLN eventually can enter the SI-LP.

## Discussion

Here, we have directly addressed how cognate T–B-cell interactions impact on Th cell subsets emigrating from a reactive LN. We show that the exit of antigen-specific CD4^+^ T cells from the LNs is coordinated such that egress of tissue-tropic effector cells precedes the appearance of Tfh-like cells in the efferent lymph. Furthermore, B cells reduce the expression of peripheral homing molecules on T cells and are critical for the appearance of lymph-borne CXCR5^+^PD-1^+^ Tfh-like cells. These latter cells are derived from Bcl6^+^ precursors in the LN and appear when the magnitude of the efferent lymph response peaks 4–5 days after immunization. At the time when the LN Tfh cell compartment has contracted to only include fully committed CXCR5^+^PD-1^high^ GC-Tfh cells, the Tfh-like cells are absent from the lymph. Th cell differentiation events driven by antigen-presenting B cells are however not restricted to increased expression of PD-1 and downmodulation of the peripheral homing markers but involve a broader transcriptional program. By using a transgenic marker for IL-4 production, we show that the expression of IL-4 was completely dependent on cognate B-cell interaction and accordingly confined to the Tfh cell lineage. By exploiting the selective and prolonged expression of a transgenic IL-4 reporter, we were able to track Tfh-like cells in the efferent lymph and as they entered the SI-LP to be integrated into the CCR7^−^CD62L^−^ effector T-cell compartment.

During recent years, B-cell depletion therapy of autoimmune diseases has put new emphasis on the role of B cells in programming T-cell responses (13, 50). The beneficial effects observed in clinical trials with anti-CD20 therapy against multiple sclerosis (MS) could, for example, not be fully ascribed to the effects on antibody responses (51). Consistent with this, an important role for antigen-presenting B cells, disconnected from humoral immunity, has been demonstrated in the murine EAE model of MS (14). While these studies imply that cognate B- and T-cell interactions have direct effects on peripheral T-cell responses and cellular immunity, loss of Tfh cells stands out as the most well-documented CD4 T-cell phenotype in µMT mice or mice with B-cell-specific deletion of MHC-II (3, 38, 45). In this regard, we show that impaired Tfh cell differentiation in mice lacking MHC-II expressing B cells goes beyond the subset of *bona fide* Tfh cells within GCs and also affects Tfh-like cells entering the circulation and intestinal tissue. A role for intestinal CXCR5^+^ Th cells in sustaining cell-mediated immunity and inflammation, most likely through secretion of IL-21, has been supported by experimental animal models as well as in expression analyses of material from patients with colorectal cancer and inflammatory bowel disease (52, 53). However, functional studies of memory and effector functions of Tfh cells extracted from tissues have been scarce. This can probably at least partially be explained by the recent finding that Tfh cells are selectively prone to NAD-induced cell death, resulting in a drastic underrepresentation of these cells in tissue preparations (54).

Both Tfh and Tfh-like cells require Bcl6 and ICOS for their generation, indicating that they are developmentally related (16, 34). Detailed flow cytometric analyses have also revealed increased expression of Bcl6 in a subset of Tfh-like cells in human blood and lymph (18, 19). In addition, several studies have demonstrated that circulatory Tfh-like cells correlate with Tfh cell differentiation in lymphoid tissues (55), vaccine responses (17, 19), and elevated autoantibody levels in systemic lupus erythematosus and rheumatoid arthritis patients (56, 57). The current finding that both the circulatory and LN-resident subsets are dependent on antigen-presenting B cells reinforces the notion that they are indeed developmentally related. This is further supported by the sustained expression of IL-4 as well as Bcl6 in Tfh-like cells in the lymph. Yet, our results show that circulatory Tfh-like cells are not derived from GC-Tfh cells as they exclusively exit the LN prior to evident GC formation. He et al. reached the same conclusion when studying mice with T cells deficient in SAP (16). While GC formation is impaired in these mice, SAP deficiency in T cells did not lead to reduced appearance of CCR7^−^CXCR5^+^PD-1^+^ Tfh-like cells in blood. Given that SAP controls the ability of T cells to make stable interactions with B cells (11), the absence of lymph-borne Tfh-like cells in mice lacking MHC-II expressing B cells seems discordant with the observation that the same cells are present in the blood when T-cell-intrinsic SAP signaling is prevented. This could reflect that more short-lived and SAP-independent interactions between B and T cells are permissive for the generation of the peripheral Tfh-like cells (but not for the GC-Tfh cells). However, as the Tfh cell-associated IL-4 production is completely lost in mice with SAP-deficient T cells (58), our finding that IL-4-producing Th cells fail to develop in the absence of MHC-II on B cells indicates that also the IL-4^+^ Tfh-like cells in the efferent lymph develop through more stable and SAP-dependent interactions with B cells.

The number of total OT-II cells in the MLN, and their frequency in the efferent lymph, was similar in the presence or absence of MHC-II molecules on B cells. Antigen-presenting B cells thus appeared to have no or minimal impact on proliferation of the OVA-specific CD4^+^ T cells. In other experimental systems, B cells have been reported to enhance T-cell proliferation (59) and even to be required for initial priming of a naive virus-like particle-specific CD4^+^ T-cell response (60). The extent by which antigen-presenting B cells impact on T-cell proliferation thus appears to depend on the nature of the antigenic challenge. It seems likely that responses where B cells make large contributions to overall T-cell expansion also manifest a T-cell differentiation program strongly influenced by the B cells. In the current study, we have primarily used the CXCR5^+^PD-1^+^ phenotype as a retrospective marker for past cognate B-cell interaction and showed that these cells constitute approximately 7%–8% of all exiting T cells at the time when LN egress peaks 4–5 days after immunization. It, however, remains possible that the impact of B cells on T-cell differentiation is not limited to this particular subset. Such a broader role for B cells in programming the Th cell response is also supported by our finding that α_4_β_7_ expression was increased on both the CXCR5^+^ and CXCR5^−^ OT-II cell subsets in mice lacking MHC-II on B cells. It can be envisioned that single-cell RNA sequencing approaches might offer a more complete understanding of the width by which B cells impact on the peripheral Th cell response. Strikingly, antigen-presenting B cells influenced the expression of gut-homing molecules only during a narrow time window. In addition, only during the same limited period of time did cognate B-cell interaction promote the generation of peripheral CXCR5^+^PD-1^+^ Tfh-like cells. We speculate that this “window of opportunity” for B cells reflects that they mostly act as secondary APCs and gain the ability to impact on the T-cell differentiation process first after significant clonal B-cell expansion has occurred. This model would support a continuum of Th cell differentiation, where Th cells upon initial interaction with DCs are programmed to develop a broad effector phenotype, including expression gut-homing receptors. This would lead to that some T cells rapidly exit the LN, while others, possibly due to low expression of molecules required for egress (61), do not. Sequentially, some T cells that remain in the LN will interact with antigen-presenting B cells in the interfollicular region. This would either lead to Th cell migration to the B-cell follicle and full GC-Tfh cell commitment or LN exit through efferent lymphatics as Tfh-like cells, of which some continue to express surface receptors mediating gut tropism.

A limitation of the study is that the potential functional roles of Tfh-like cells in the TDL and intestinal preparation could not be addressed due to difficulties in isolating sufficient cell numbers from these tissues. In addition, the choice of i.p. rather than oral immunizations when studying Th activation in the MLN and α_4_β_7_ expressing TDLs could also be considered a limitation. However, i.p. immunizations render less variability in T-cell expansion in the MLNs compared with feeding antigen with adjuvants, which would have required increased numbers of cannulations to be performed. Importantly, our finding that almost 80%–90% of OT-II TDLs day 2.5 post-immunization express α_4_β_7_, an integrin that can only be induced by mucosal DC, support that the T cells studied (including the Tfh-like cells) primarily were primed in the MLN or PP following the i.p. immunization regimen. Not being able to directly compare the total numbers of OT-II cells in TDLs and MLN represents another limitation of the study. Due to the large variation in lymph flow between individual mice, cannulation of a very large number of mice would however have been required for this comparison, which was not possible for ethical and technical reasons. However, as the comparisons were made between OT-II cell frequencies of total CD4 T cells, which were rather constant in the two tissue preparations, and the total numbers of leukocytes did not differ between these preparations more than about 10-fold at most (MLN 10–20 × 10^6^ cells), our results do not indicate that the output of OT-II cells into the lymph represents an insignificant fraction of the OT-II cells expanding in the MLN.

In summary, we have demonstrated that circulatory Tfh-like cells similar to GC-Tfh cells fail to develop in the absence of cognate B- and T-cell interactions. Our results show that B cells program the efferent Th cell response only during a limited period of time after antigenic challenge. The ability of B cells to promote the Tfh cell lineage occurs at the expense of sustained generation of classical peripheral Teff cells. Still, downmodulation of peripheral homing molecules by B cells is incomplete, leading to LN egress of Tfh-like cells with the ability to enter peripheral tissues (here studied in the context of α_4_β_7_^+^ Tfh cells with gut-homing capacity). How B cells program the peripheral pool of Tfh-like cells should therefore not only be considered in the context of secondary GC responses but should also be taken into account when studying protective and autoimmune Th cell responses in all tissues.

## Data Availability Statement

The datasets presented in this study can be found in online repositories. The names of the repository/repositories and accession number(s) can be found below: https://www.ebi.ac.uk/arrayexpress/, E-MTAB-11165.

## Ethics Statement

The animal study was reviewed and approved by the Ethical Committee for Laboratory Animals in Gothenburg and the Ethical Committee for Laboratory Animals in Lund.

## Author Contributions

Conceptualization: SA, BJ-L, and UY. Formal analysis: SA, LS, and JK. Investigation: SA, JC, LS, YD, UW, JK, HC, and ML. Data curation: SA, LS, YD, and JK. Writing, review, and editing: SA, DB, BJ-L, and UY. Supervision: QL, BJ-L, and UY. All authors have read and agreed to the submitted version of the manuscript.

## Funding

This research was funded by grants from the Swedish Research Council (2017-02646) to UY and from the Swedish Cancerfonden (18 0324) and the Lundbeck Foundation (R155-2014-4184) to BJ-L.

## Conflict of Interest

The authors declare that the research was conducted in the absence of any commercial or financial relationships that could be construed as a potential conflict of interest.

## Publisher’s Note

All claims expressed in this article are solely those of the authors and do not necessarily represent those of their affiliated organizations, or those of the publisher, the editors and the reviewers. Any product that may be evaluated in this article, or claim that may be made by its manufacturer, is not guaranteed or endorsed by the publisher.
