# Supplementary material for: Antigen-Presenting B Cells Program the Efferent Lymph T Helper Cell Response
Source: Front Immunol. 2022 Mar 9;13:813203. doi: 10.3389/fimmu.2022.813203 (PMC8959485; doi:10.3389/fimmu.2022.813203)
Supplement: Supplementary file 1 [file Presentation_1.pdf]

Supplementary Figure 1

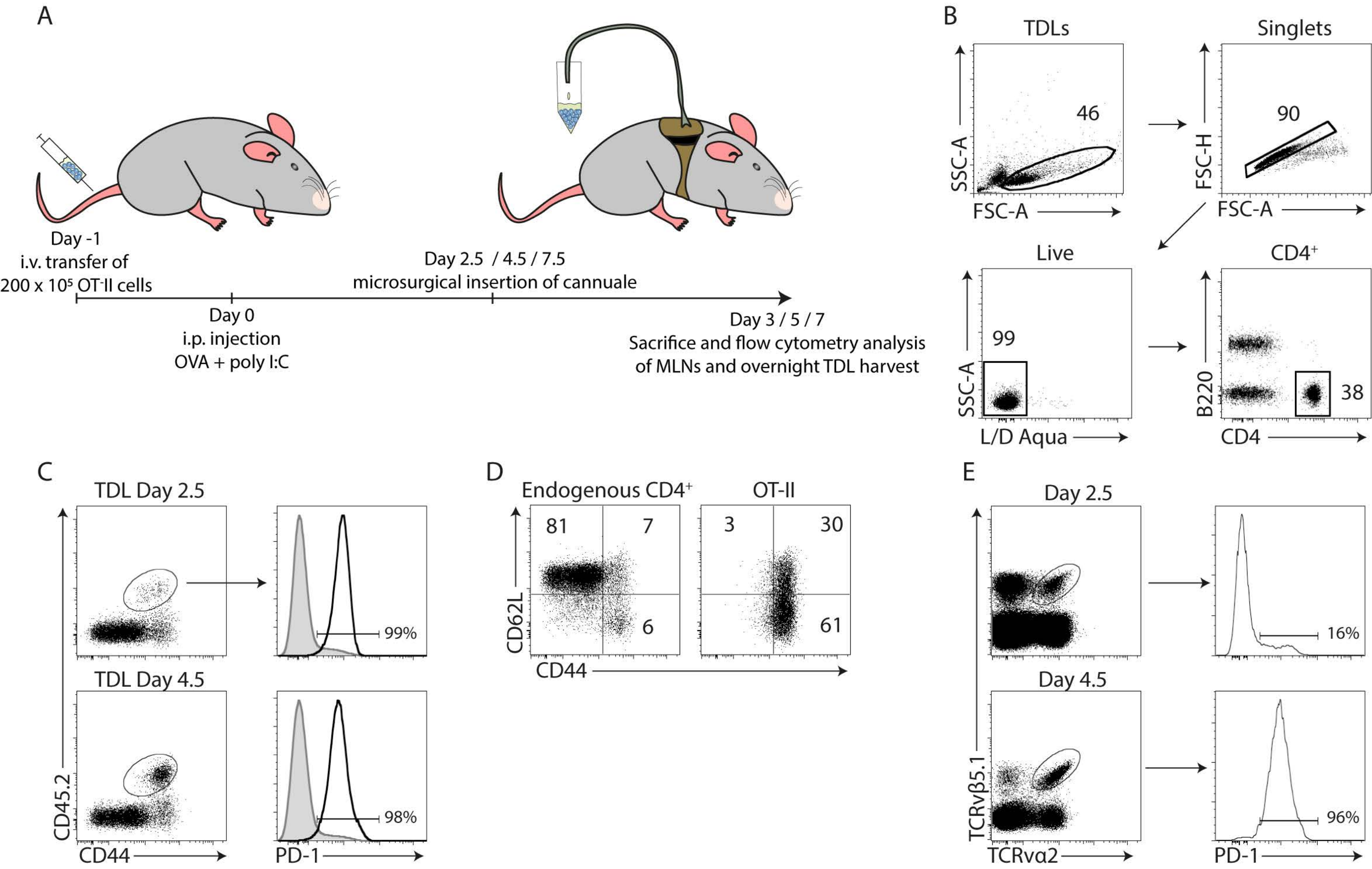

Supplementary Figure 1. Ag-specific Th cells in efferent lymph display an activated phenotype at early time points after immunisation. (A-C) CD45.1⁺ OT-II cells were transferred into congenic CD45.2⁺ hosts one day prior to i.p. immunization with OVA + polyI:C and TDLs were collected and analysed by flow cytometry at indicated intervals. (A) Schematic overview of the experimental procedure. (B) Dot plots showing gating strategy to identify CD4⁺ T cells in MLN and TDL. (C) Representative flow cytometry plots of CD45.2 and CD44 expression used to identify transferred OT-II cells and the expression of PD-1 by transferred cells (line) and endogenous T cells (grey) (D) Representative flow cytometry plots of CD62L and CD44 expression by endogenous T cells and CD45.2⁺ OT-II cells in TDL 96-120h p.i. (E) Representative flow cytometry plots of PD-1 expression by T cells identified by TCRvβ5.1 and TCRvα2 expression. Data representative from at least two independent experiments.

Supplementary Figure 2

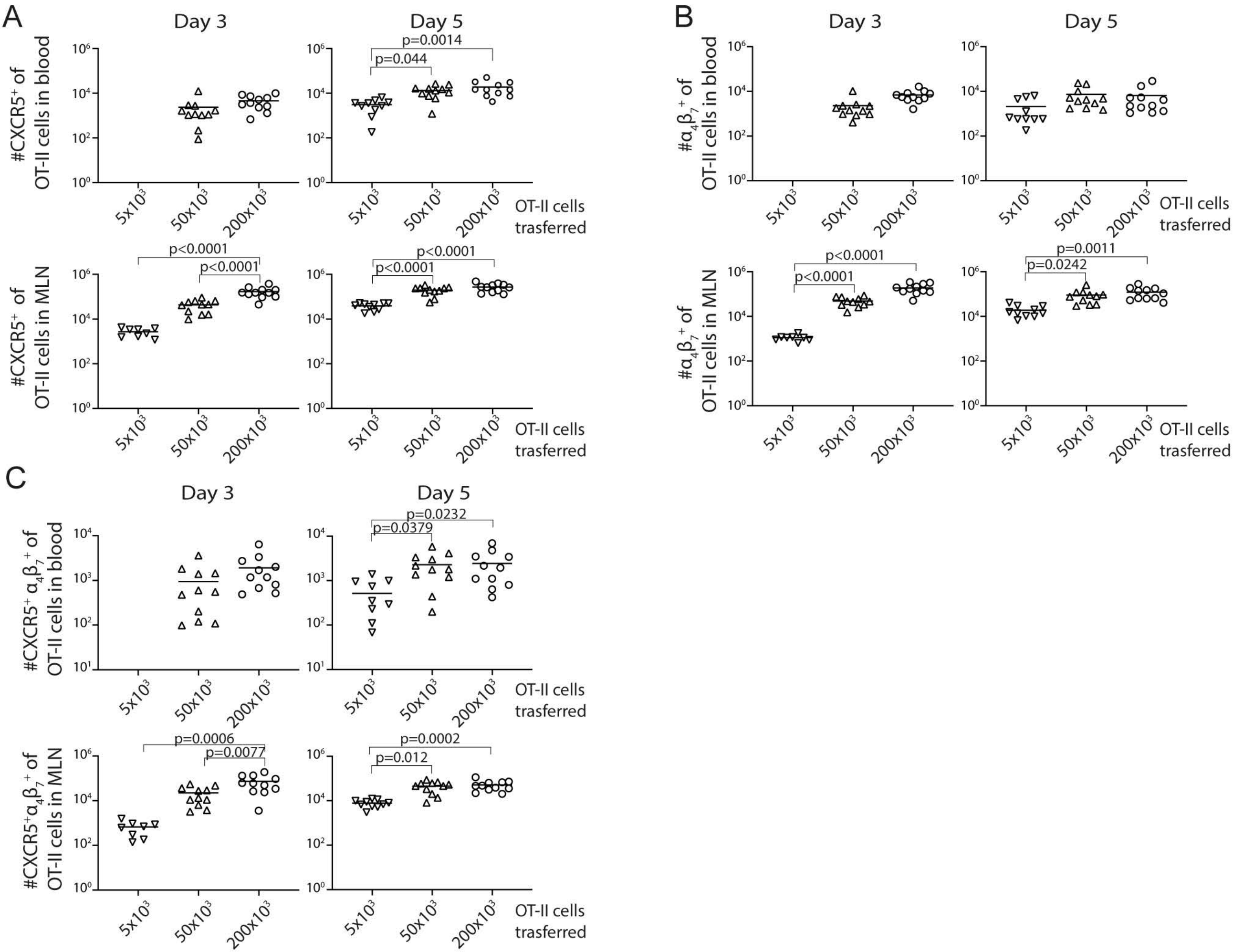

Supplementary Figure 2. Increased number of precursor cells leads to increased generation of CXCR5 and $\alpha_4\beta_7$ expressing OT-II cells following immunization. (A-C) Indicated numbers of OT-II cells were adoptively transferred and enumeration of CXCR5$^+$ (A), $\alpha_4\beta_7^+$ (B) and CXCR5$^+$ $\alpha_4\beta_7^+$ (C) OT-II cells in blood and MLN at three- and five-days post immunization is presented in bar graphs. Pooled data from three independent experiments, n=8-11 per group. One-way ANOVA with Tukey´s multiple comparison test, p-values <0.05 are reported.

Supplementary Figure 3

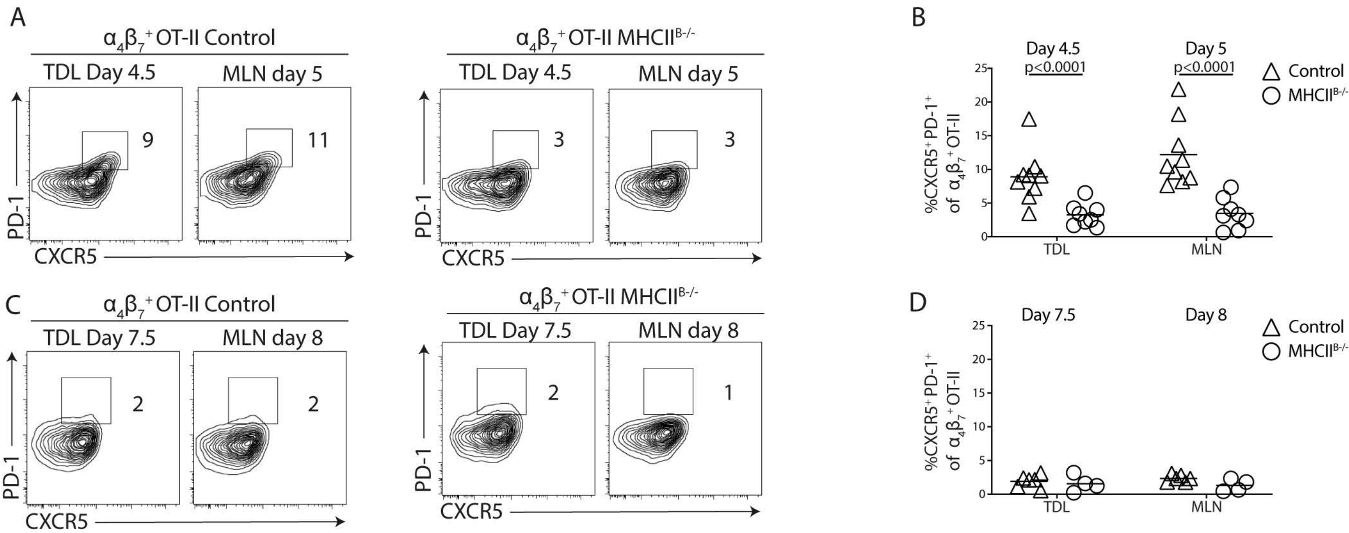

Supplementary Figure 3. Cognate B cell interaction is required for appearance of $\alpha_4\beta_7^+$ Tfh-like cells in efferent lymph. CD45.1$^+$ OT-II cells were transferred into control or MHCII$^{B-/-}$ chimeras that were immunized with OVA + polyI:C the following day. TDLs were collected at indicated intervals prior to sacrifice and harvest of MLN 5 or 8 days p.i. for parallel flow cytometry analyses. (A-B) Representative flow cytometry analysis of CXCR5 and PD-1 expression on $\alpha_4\beta_7^+$ OT-II cells among TDLs and in MLNs (A) and percentages of CXCR5$^+$PD-1$^+$ among $\alpha_4\beta_7^+$ OT-II cells (B) at 4.5 and 5 days post-immunization. (C-D) Flow cytometry plots (C) and percentages of CXCR5$^+$PD-1$^+$ among $\alpha_4\beta_7^+$ OT-II cells at (D) day 7.5 and 8. (A and B) represent two individual experiments, n=4-6. (C and D) two individual experiments, n=7-9 and. Two-tailed unpaired student's t-test of one-way ANOVA with Sidak's multiple comparison test was used for statistical analysis, p-values <0.05 are reported.

Supplementary Figure 4

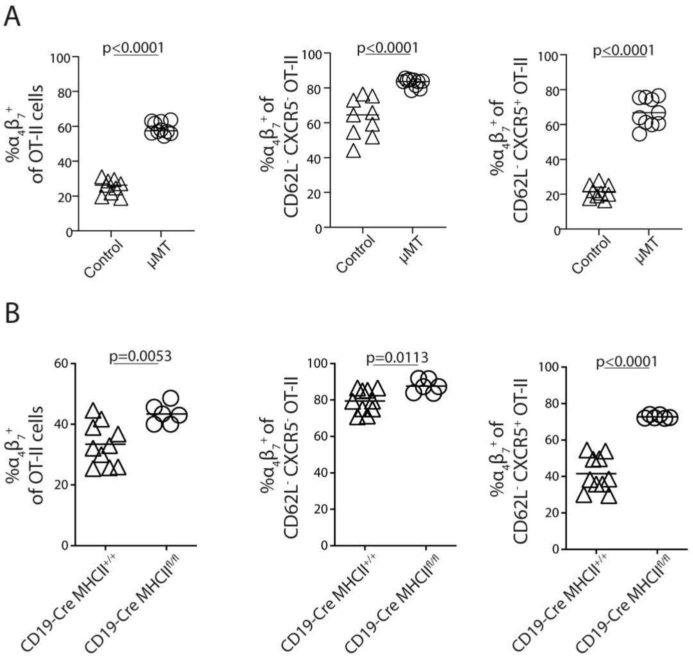

Supplementary Figure 4. Increased generation of $\alpha_4 \beta_7^+$ Th cells in MLNs in the absence of cognate T-B interactions. (A-B) CD45.1$^+$ OT-II cells were transferred into control and μMT or CD19-CreMHCII$^{fl/fl}$ and CD19-CreMHCII$^{+/+}$ controls that were immunized with OVA + polyI:C the following day. MLNs were collected 5 days p.i. for flow cytometry analyses. (A) Percentage of $\alpha_4\beta_7^+$ cells among OT-II cells and frequency of $\alpha_4\beta_7^+$ cells among CD62L$^-$ CXCR5$^-$ and CD62L$^-$ CXCR5$^+$ OT-II cells in MLNs of control and μ MT mice pooled from two independent experiments, n=9/10. (B) Identical analyses as in (A) were performed in CD19-CreMHCII$^{fl/fl}$ and CD19-CreMHCII$^{+/+}$ controls and pooled from two independent experiments, n=10/6. For statistical analysis , unpaired two-tailed student's t-test was used, p-values <0.05 are reported.

Supplementary Figure 5

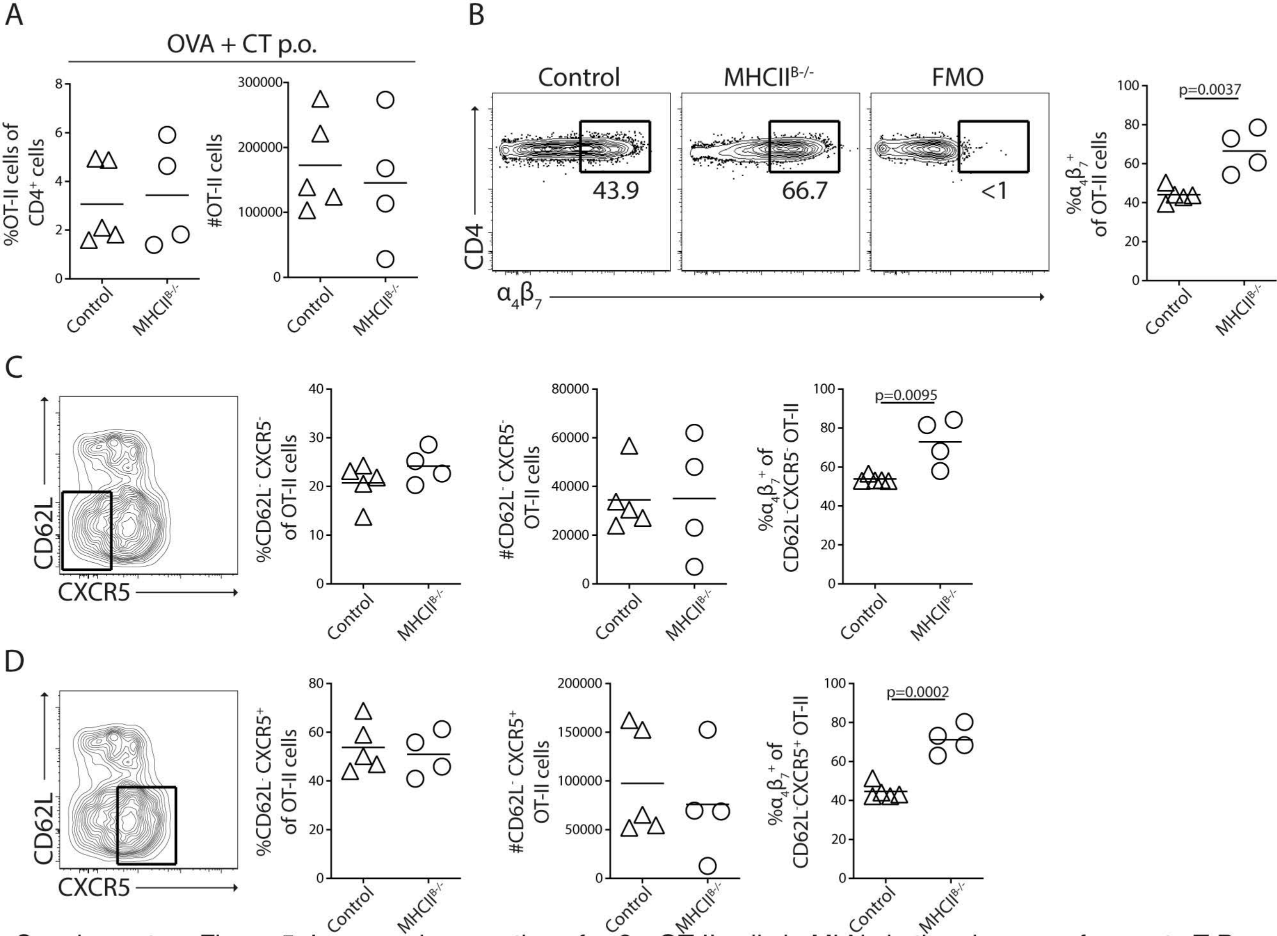

Supplementary Figure 5. Increased generation of $\alpha_4\beta_7^+$ OT-II cells in MLNs in the absence of cognate T-B interactions is not dependent on adjuvant and route of administration. (A-D) $CD45.1^+$ OT-II cells were transferred into control or $MHCII^{B-/-}$ chimeras that were immunized with 3 mg OVA and 10 µg CT orally the following day. MLNs were collected 5 days p.i. for flow cytometry analyses. (A) Percentage of OT-II cells among $CD4^+$ T cells as well as number OT-II cells in control and $MHCII^{B-/-}$ mice. (B) Representative flow cytometry analysis of $\alpha_4\beta_7$ by OT-II cells and percentage of $\alpha_4\beta_7^+$ among OT-II cells in control and $MHCII^{B-/-}$ mice. (C-D) Gating of (C) $CD62L^-$ $CXCR5^-$ and (D) $CD62L^-$ $CXCR5^+$ subset of OT-II cells. Percentage of the respective subset of OT-II cells, total numbers of the subsets and frequency of $\%\alpha_4\beta_7^+$ in each subset in control and $MHCII^{B-/-}$ mice gated as in (B). Data from one experiment, n=5/4. Statistical analysis was performed using unpaired two-tailed student's t-test, p-values <0.05 are reported.

Supplementary Figure 6

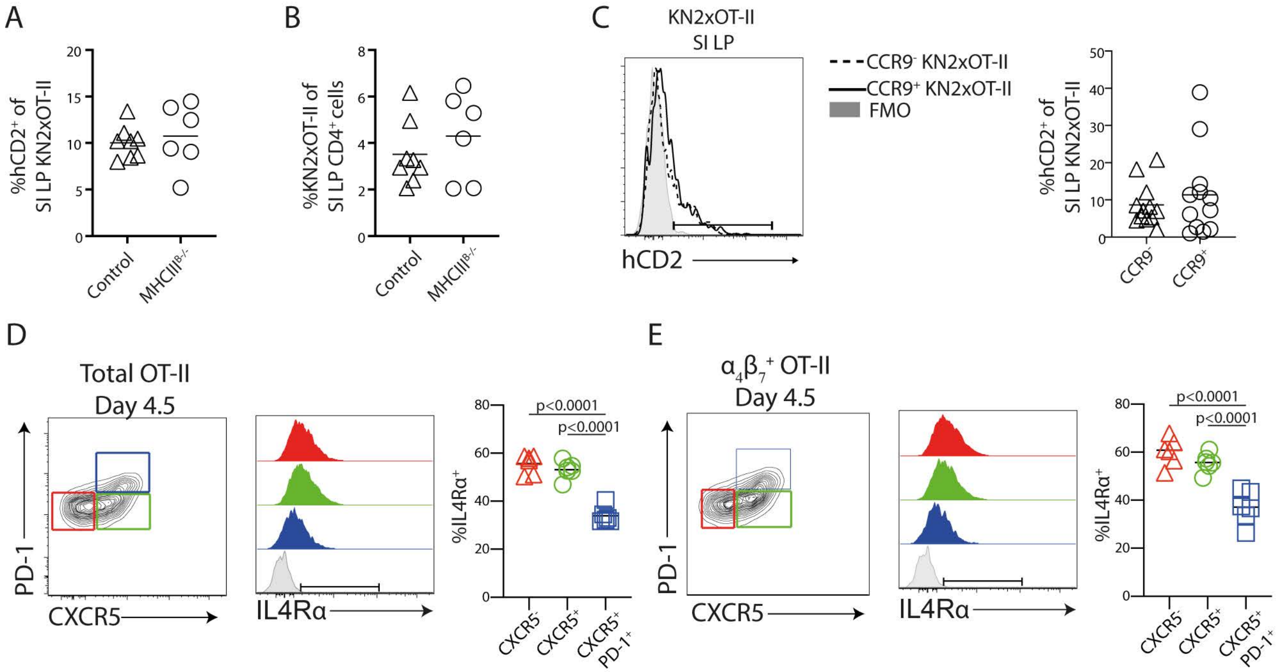

Supplementary Figure 6. Transgenic hCD2 expression by OT-II cells in SI LP and IL4Rα expression by OT-II TDLs. (A-C) KN2xOT-II cells were transferred into control or MHCII$^{B-/-}$ chimeras (A-B) or wild type mice (C) which were the next day immunized with OVA and polyI:C. Five days later mice were sacrificed and SI LP cells were isolated for flow cytometry analyses. Percentage of KN2xOT-II expressing hCD2 (A) and frequency of KN2xOT-II among CD4^{+} cells (B) in SI LP pooled from two independent experiments where n=8/6. (C) Representative histogram of hCD2 expression in CCR9^{+} (line) and CCR9^{-} (dashed) KN2xOT-II cells in wild type mice. Percentage of hCD2^{+} cells among CCR9$^{+/-}$ KN2xOT-II cells pooled from three independent experiments, n=12 per group. (D-E) OT-II cells were transferred into wild type mice that were immunized with OVA + polyI:C the following day. TDLs were collected at indicated time points. Representative flow cytometric analyses used to identify CXCR5^{-}, CXCR5^{+} and CXCR5^{+} PD-1^{+} subpopulations.among total OT-II cells (D) or $\alpha_4\beta_7^{+}$ OT-II cells (E). (D-E) Representative histograms of IL4Rα expression by each gated subset and percentage of IL4Rα^{+} cells of each subset pooled from two individual experiments where n=6. Unpaired two-tailed student's t-test (A-C) or one-way ANOVA was used for statistical analysis, p-values <0.05 are reported.
